# Supplementary figures and images for: A Spatial Framework for Understanding Population Structure and Admixture
Source: PLoS Genet. 2016 Jan 15;12(1):e1005703. doi: 10.1371/journal.pgen.1005703 (PMC4714911; doi:10.1371/journal.pgen.1005703)

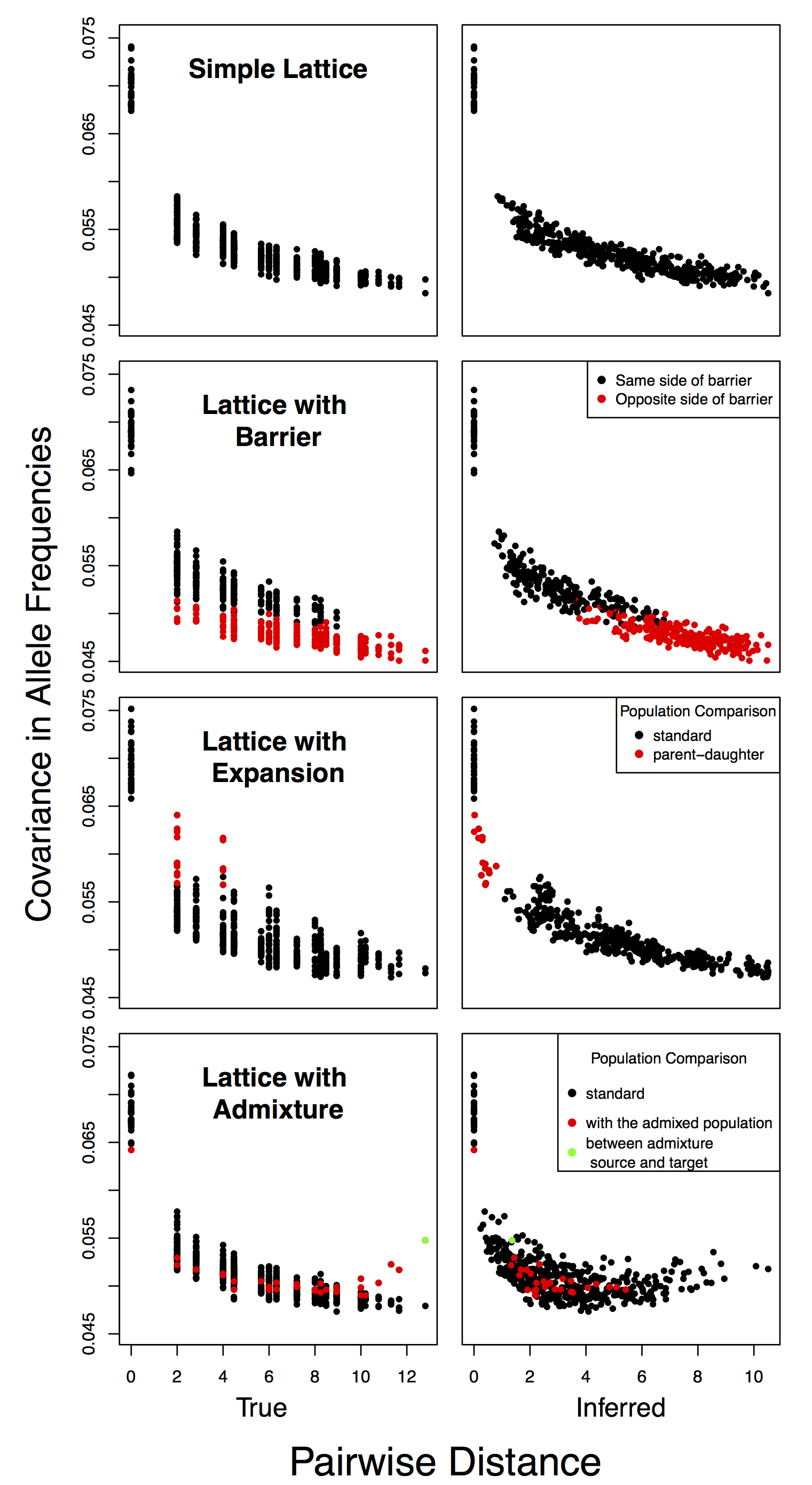

Supplement: S1 Fig — Left column: sample covariance plotted against observed pairwise distance. Right column: sample covariance plotted against inferred geogenetic distance. (TIF) [file pgen.1005703.s001.tif]

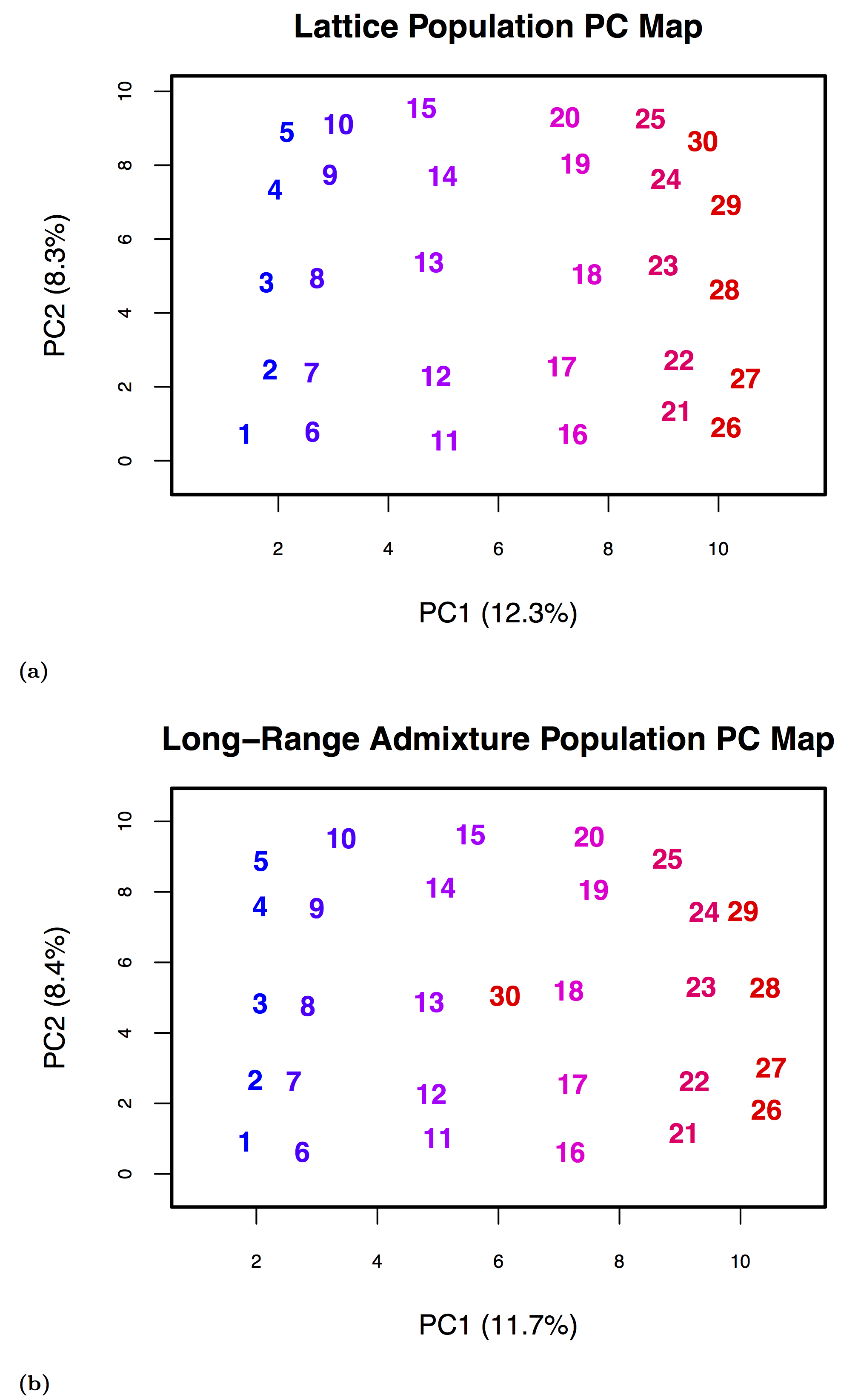

Supplement: S2 Fig — a) the basic lattice scenario shown in Fig 1a. b) the lattice scenario with admixture from Population 1 into Population 30, shown in Fig 2a. (TIF) [file pgen.1005703.s002.tif]

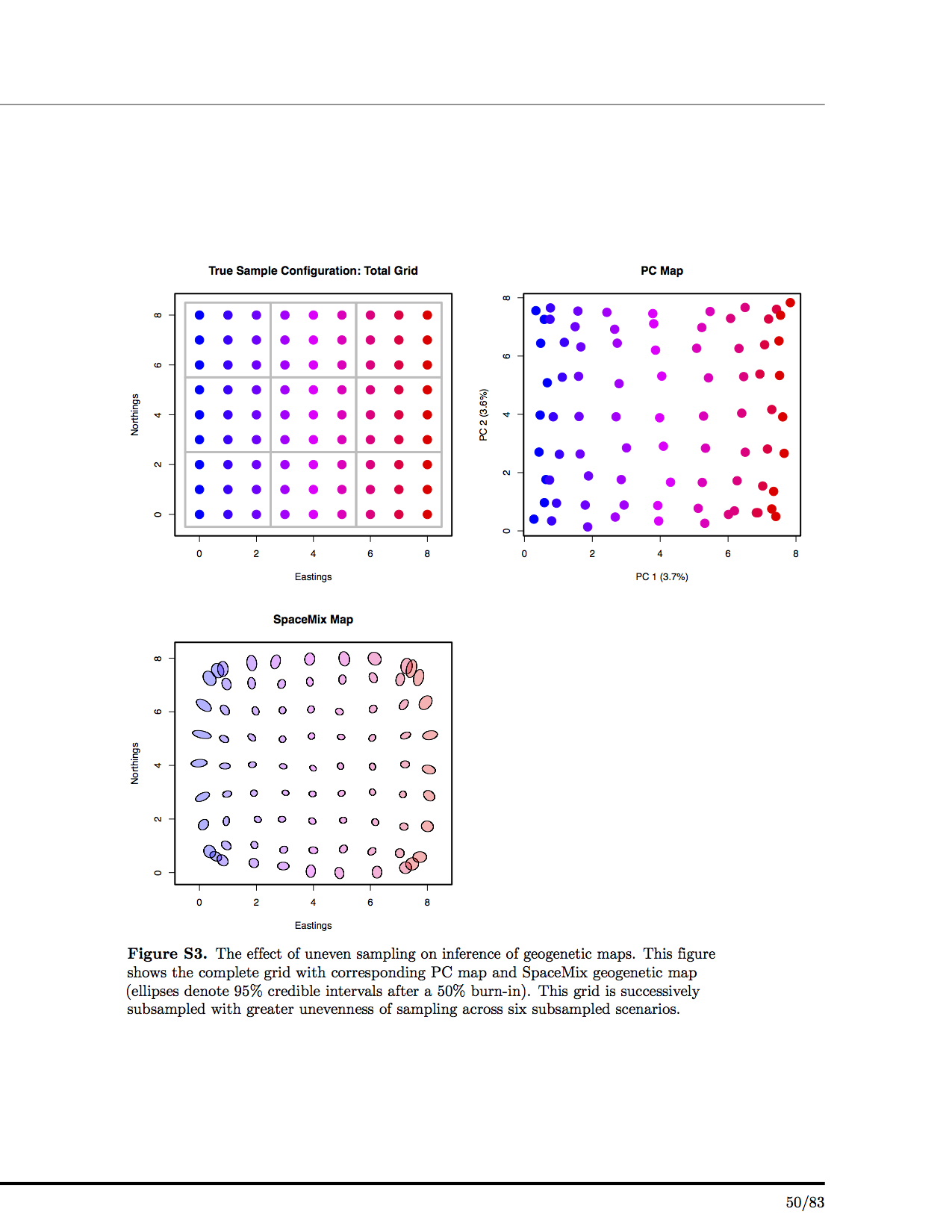

Supplement: S3 Fig — This figure shows the complete grid with corresponding PC map and SpaceMix geogenetic map (ellipses denote 95% credible intervals after a 50% burn-in). This grid is successively subsampled with greater unevenness of sampling across six subsampled scenarios. (TIF) [file pgen.1005703.s003.tif]

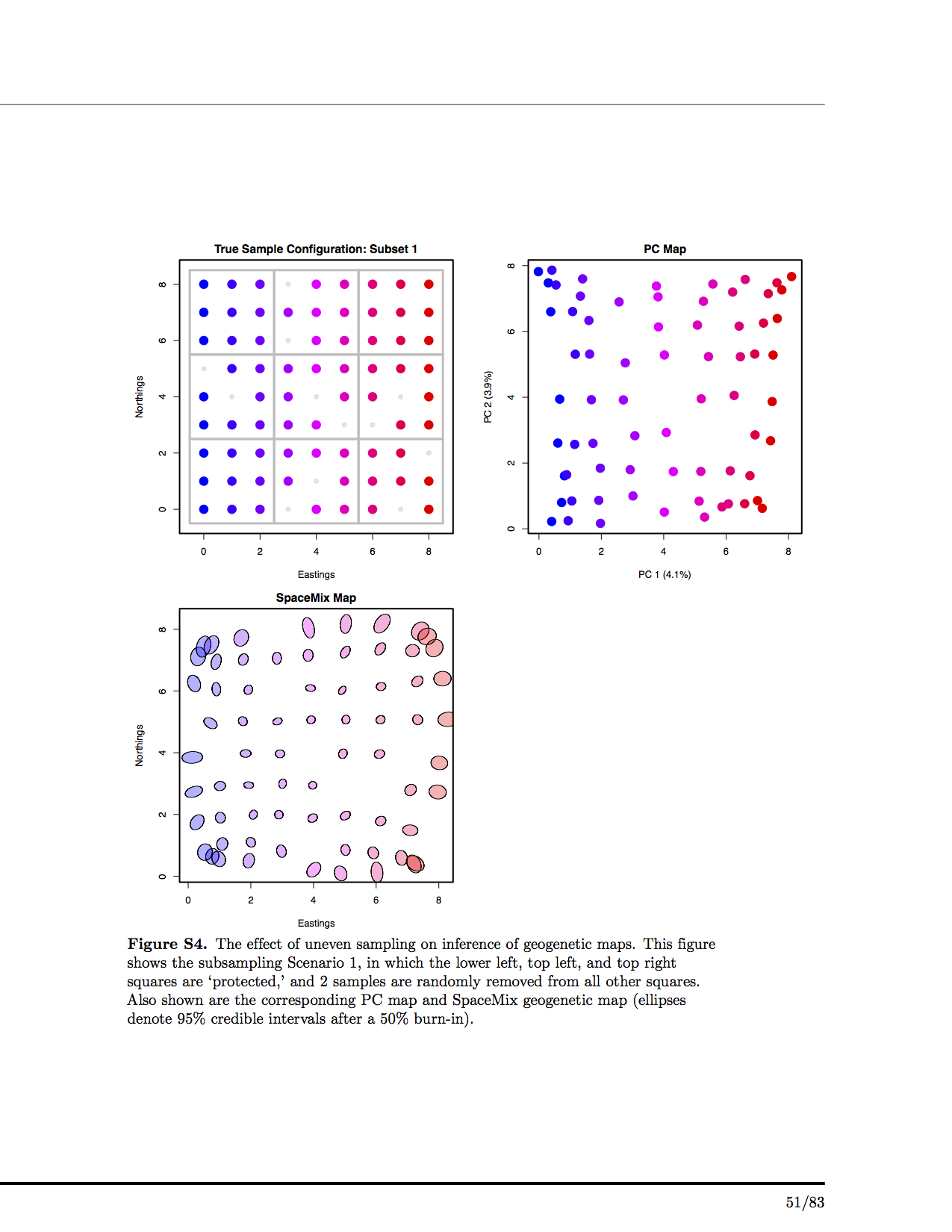

Supplement: S4 Fig — This figure shows the subsampling Scenario 1, in which the lower left, top left, and top right squares are ‘protected,’ and 2 samples are randomly removed from all other squares. Also shown are the corresponding PC map and SpaceMix geogenetic map (ellipses denote 95% credible intervals after a 50% burn-in). (TIF) [file pgen.1005703.s004.tif]

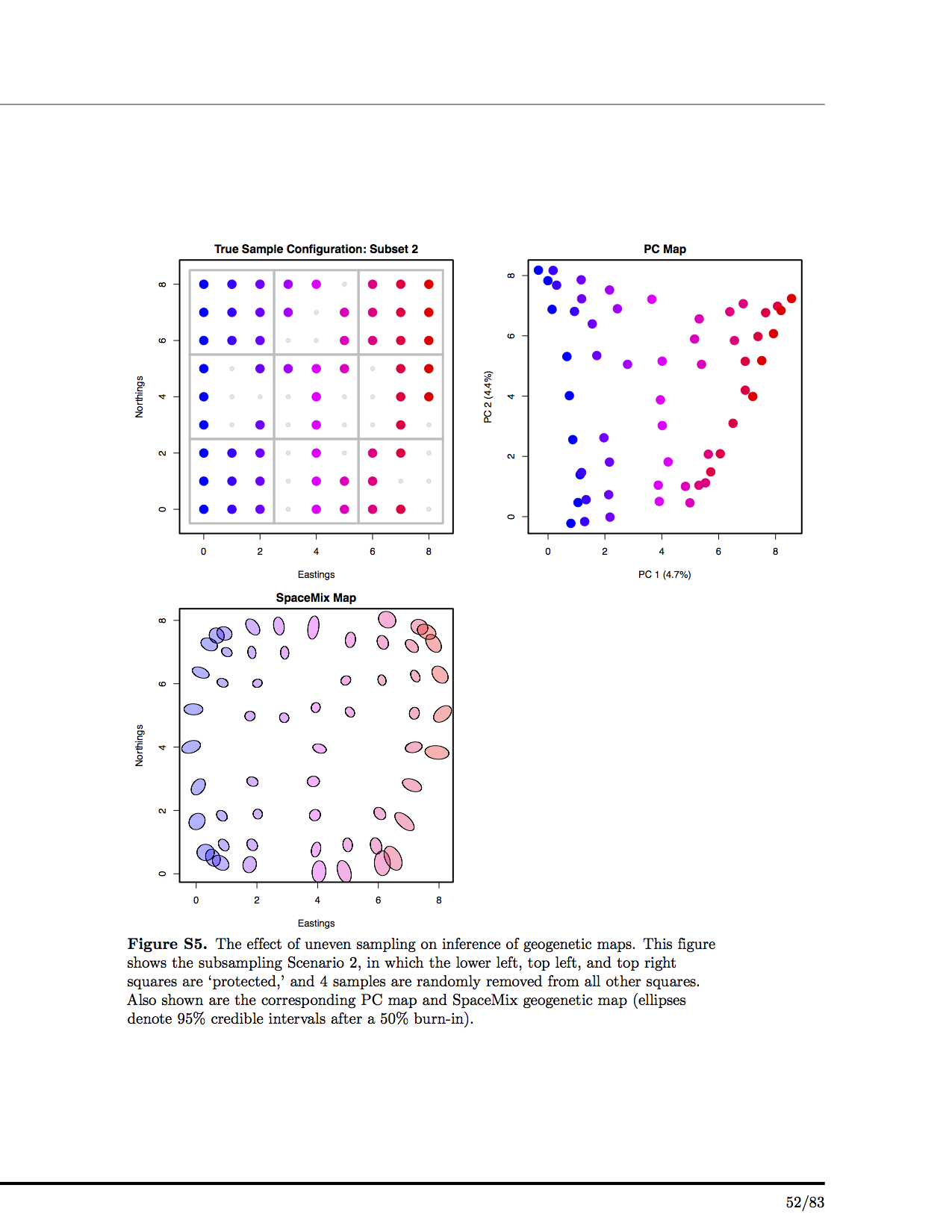

Supplement: S5 Fig — This figure shows the subsampling Scenario 2, in which the lower left, top left, and top right squares are ‘protected,’ and 4 samples are randomly removed from all other squares. Also shown are the corresponding PC map and SpaceMix geogenetic map (ellipses denote 95% credible intervals after a 50% burn-in). (TIF) [file pgen.1005703.s005.tif]

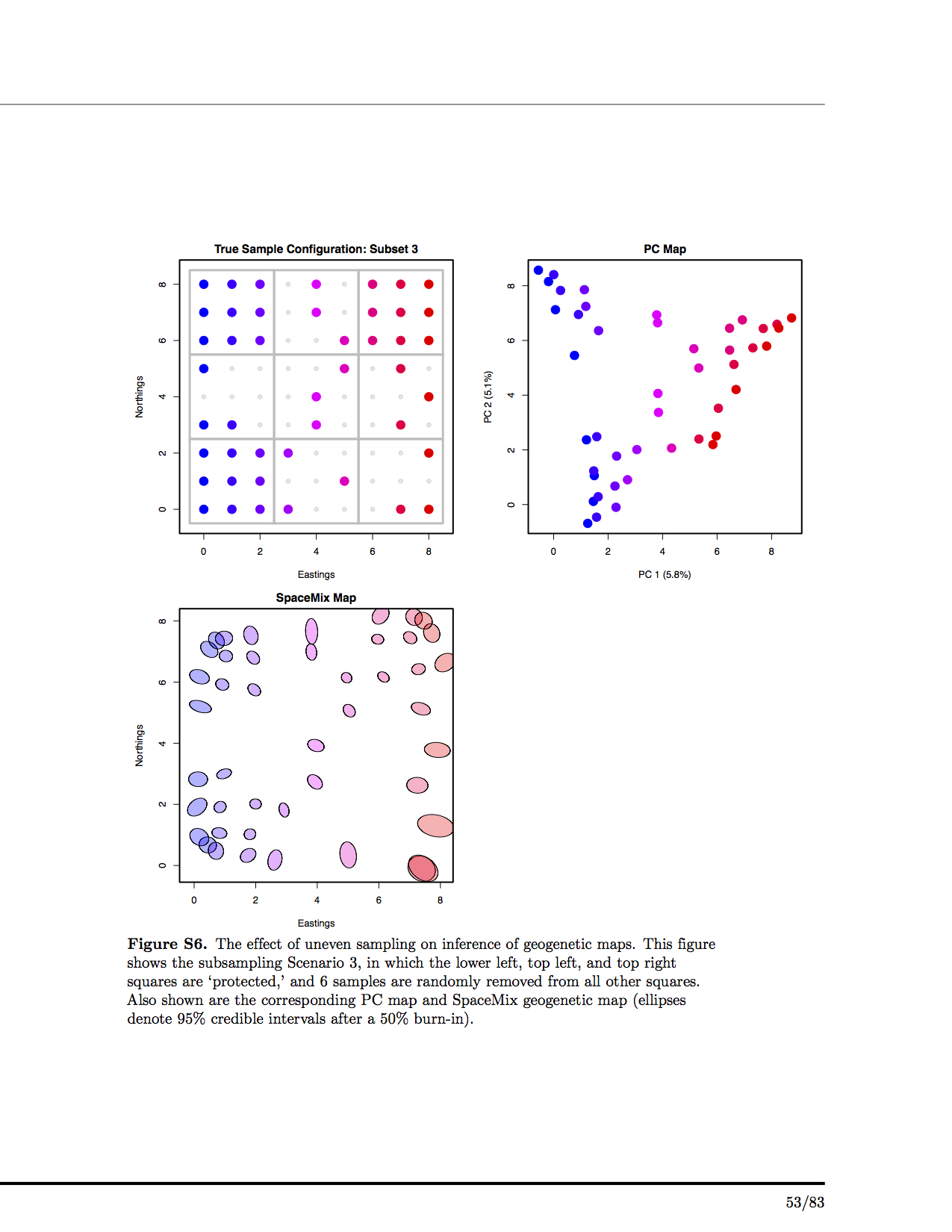

Supplement: S6 Fig — The effect of uneven sampling on inference of geogenetic maps. This figure shows the subsampling Scenario 3, in which the lower left, top left, and top right squares are ‘protected,’ and 6 samples are randomly removed from all other squares. Also shown are the corresponding PC map and SpaceMix geogenetic map (ellipses denote 95% credible intervals after a 50% burn-in). (TIF) [file pgen.1005703.s006.tif]

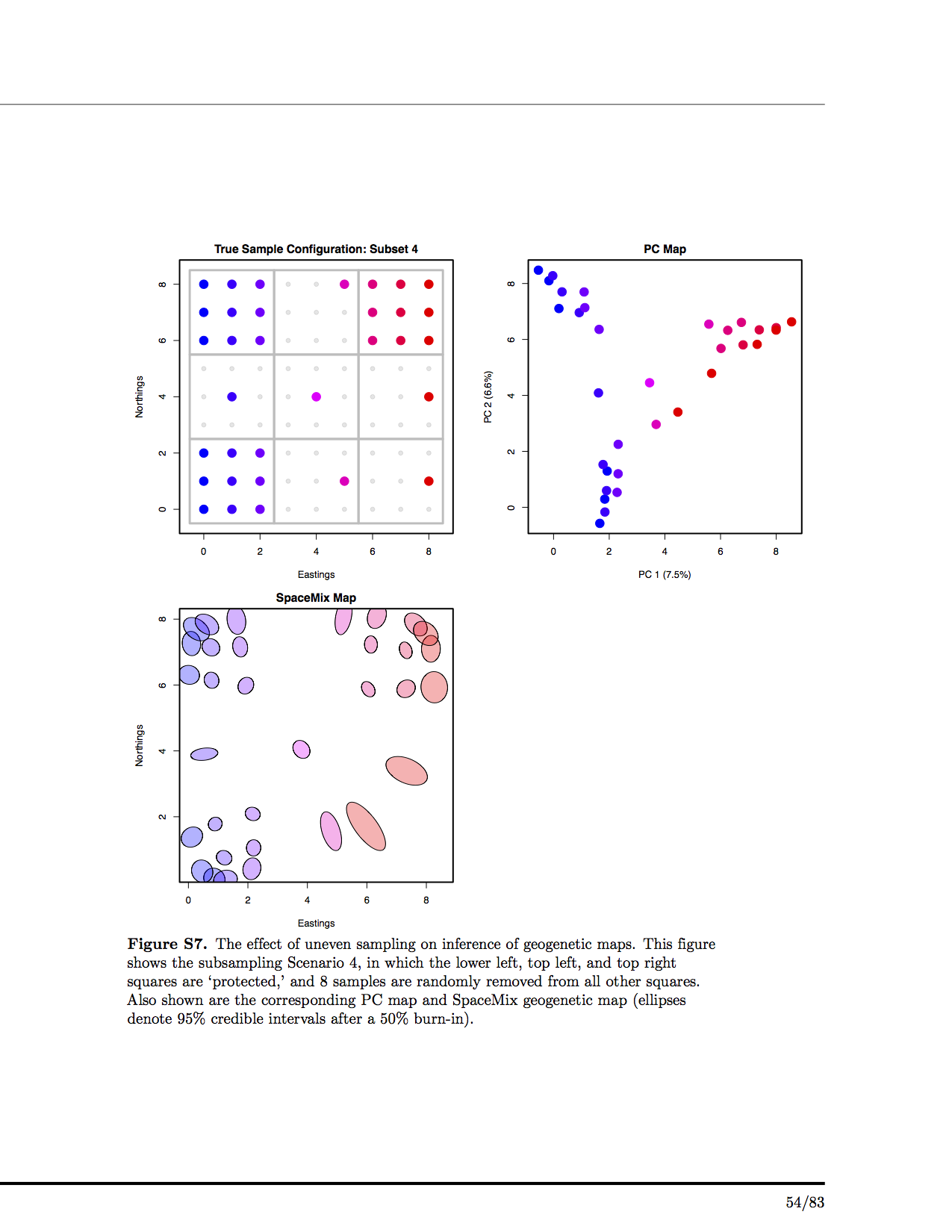

Supplement: S7 Fig — The effect of uneven sampling on inference of geogenetic maps. This figure shows the subsampling Scenario 4, in which the lower left, top left, and top right squares are ‘protected,’ and 8 samples are randomly removed from all other squares. Also shown are the corresponding PC map and SpaceMix geogenetic map (ellipses denote 95% credible intervals after a 50% burn-in). (TIF) [file pgen.1005703.s007.tif]

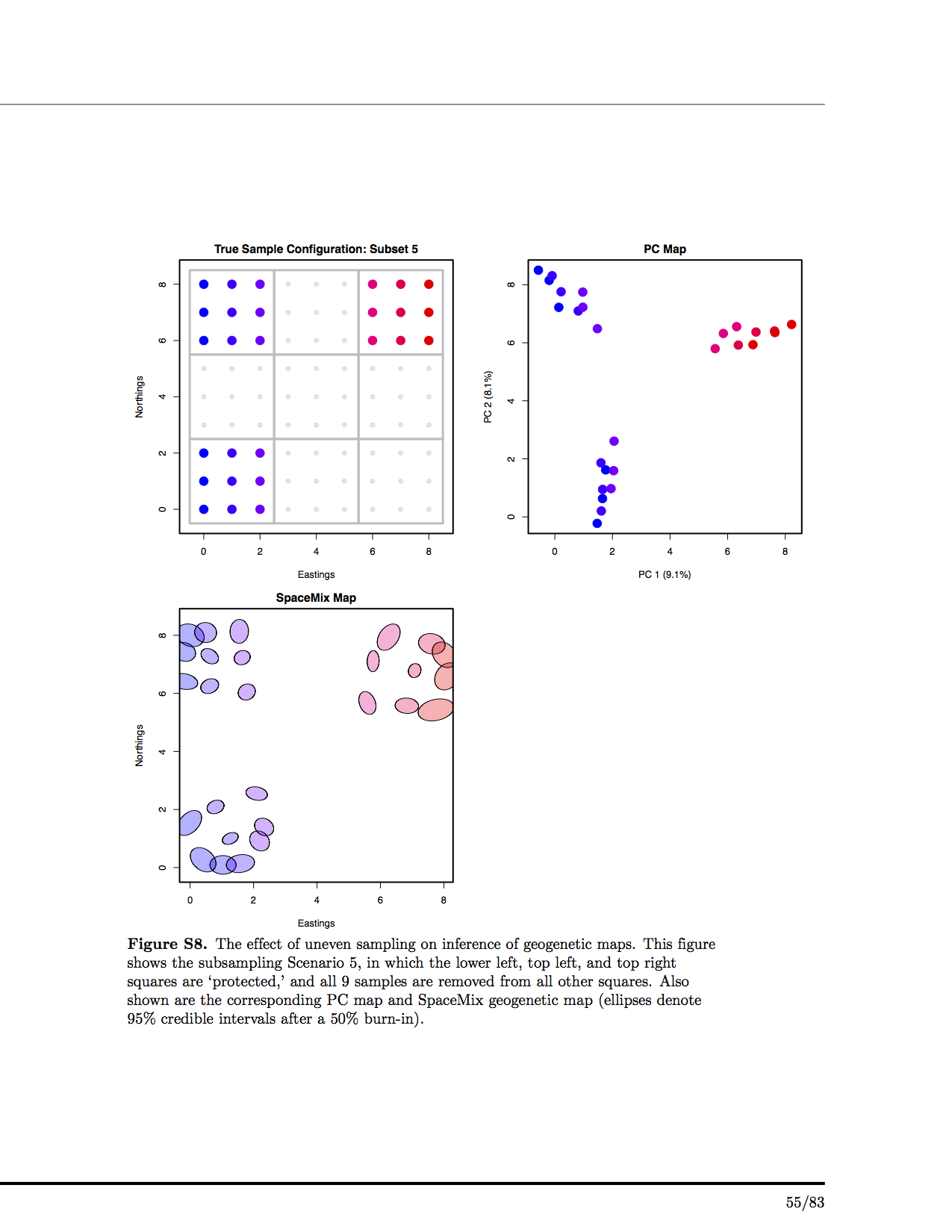

Supplement: S8 Fig — The effect of uneven sampling on inference of geogenetic maps. This figure shows the subsampling Scenario 5, in which the lower left, top left, and top right squares are ‘protected,’ and all 9 samples are removed from all other squares. Also shown are the corresponding PC map and SpaceMix geogenetic map (ellipses denote 95% credible intervals after a 50% burn-in). (TIF) [file pgen.1005703.s008.tif]

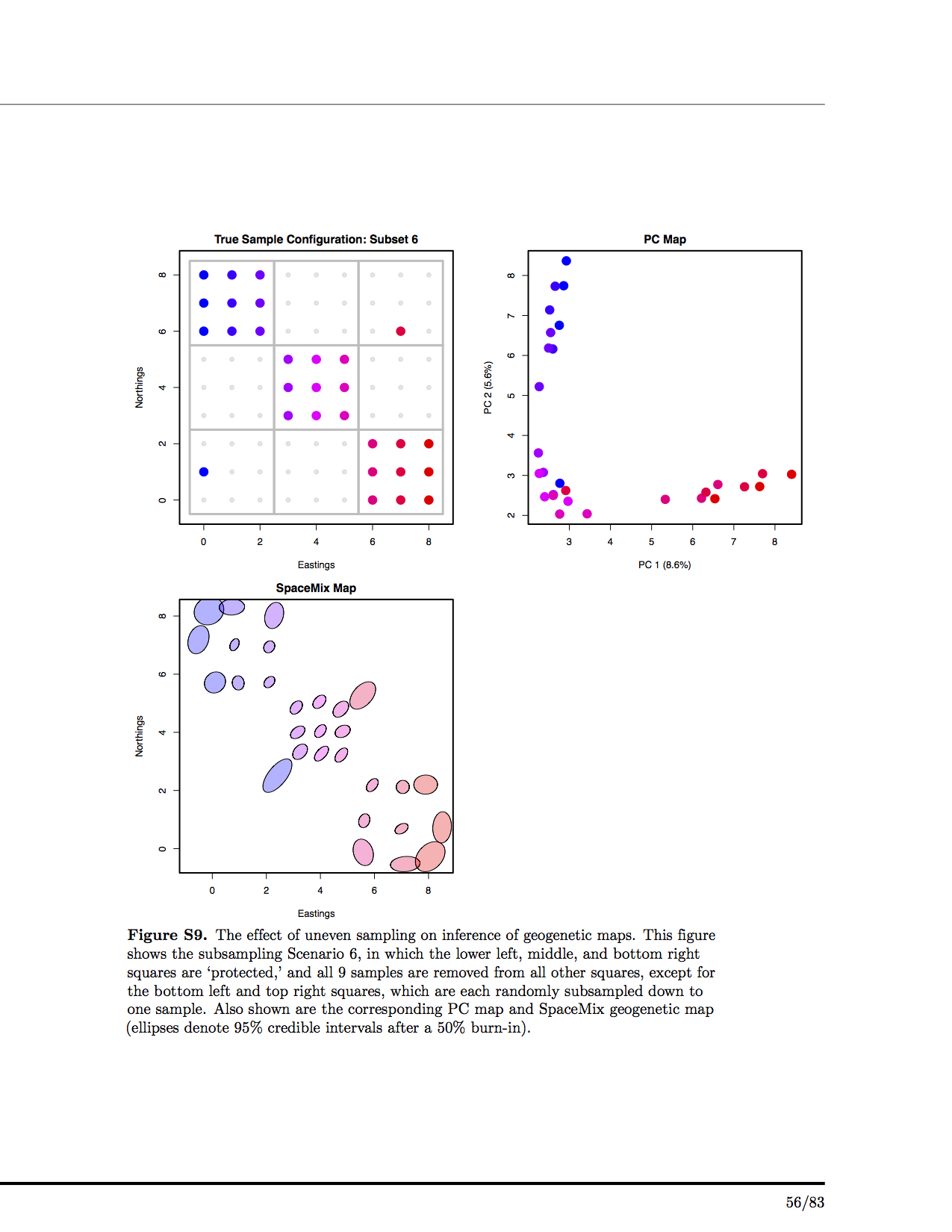

Supplement: S9 Fig — The effect of uneven sampling on inference of geogenetic maps. This figure shows the subsampling Scenario 6, in which the lower left, middle, and bottom right squares are ‘protected,’ and all 9 samples are removed from all other squares, except for the bottom left and top right squares, which are each randomly subsampled down to one sample. Also shown are the corresponding PC map and SpaceMix geogenetic map (ellipses denote 95% credible intervals after a 50% burn-in). (TIF) [file pgen.1005703.s009.tif]

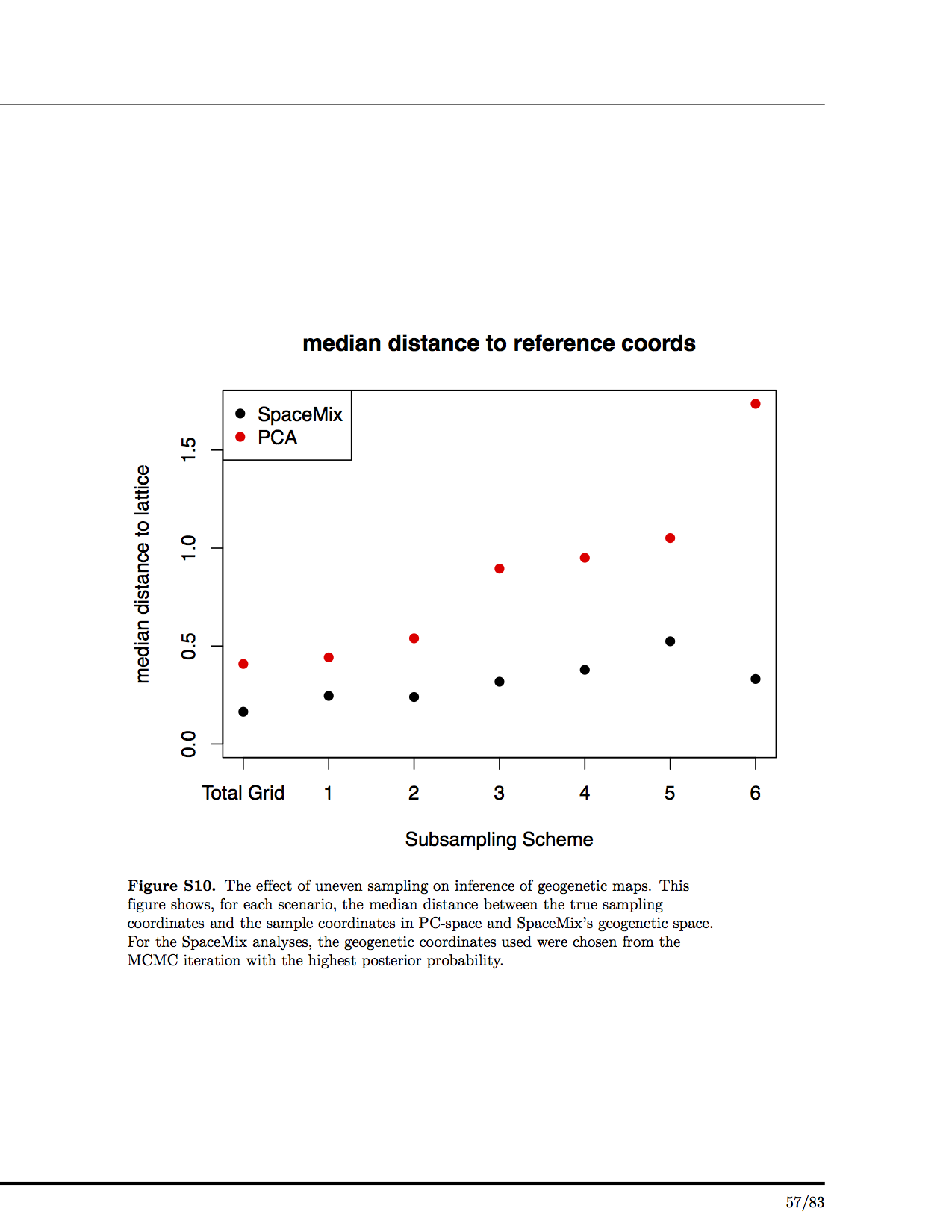

Supplement: S10 Fig — The effect of uneven sampling on inference of geogenetic maps. This figure shows, for each scenario, the median distance between the true sampling coordinates and the sample coordinates in PC-space and SpaceMix’s geogenetic space. For the SpaceMix analyses, the geogenetic coordinates used were chosen from the MCMC iteration with the highest posterior probability. (TIF) [file pgen.1005703.s010.tif]

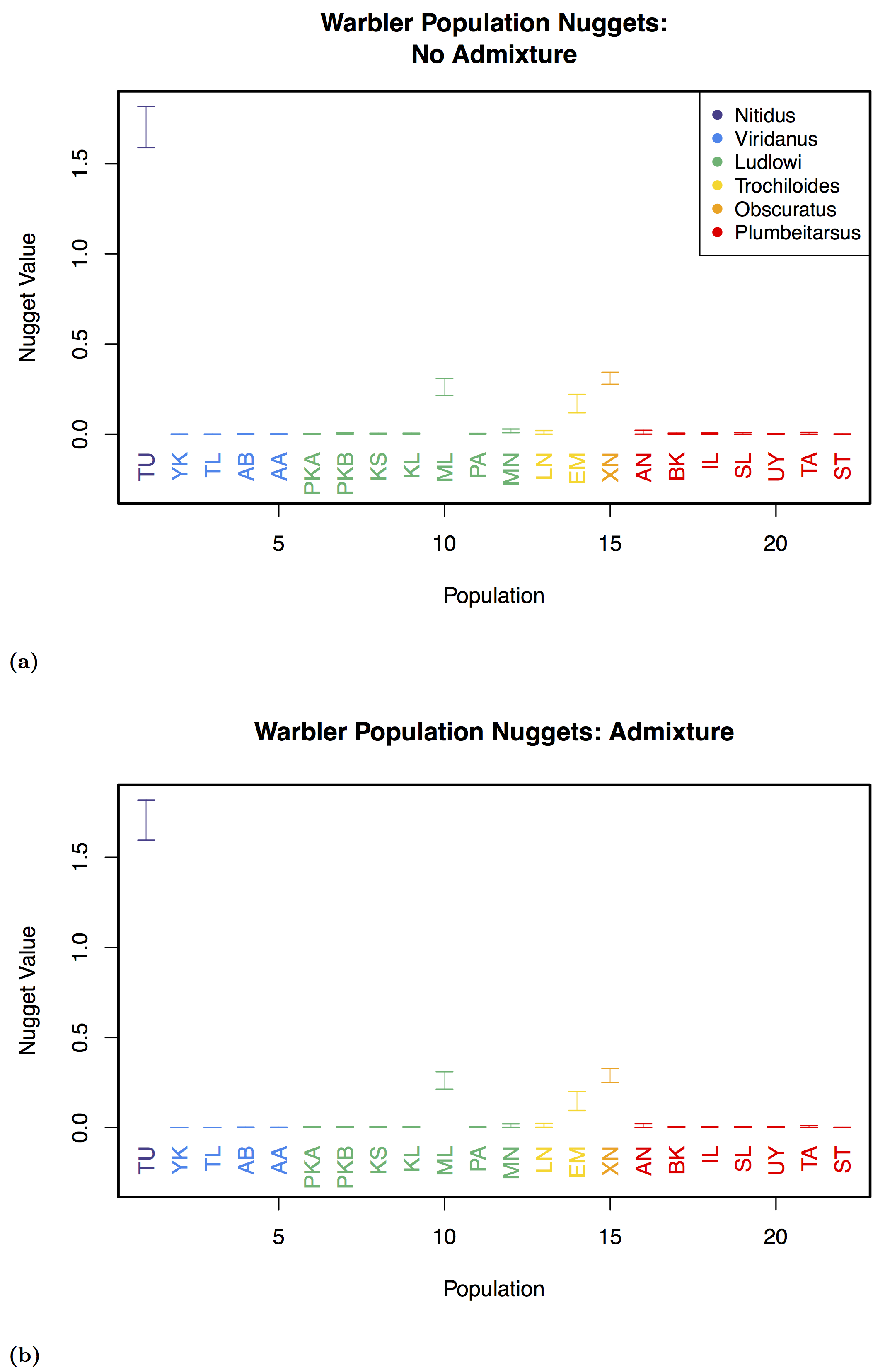

Supplement: S11 Fig — a) analysis without admixture b) analysis with admixture. (TIF) [file pgen.1005703.s011.tif]

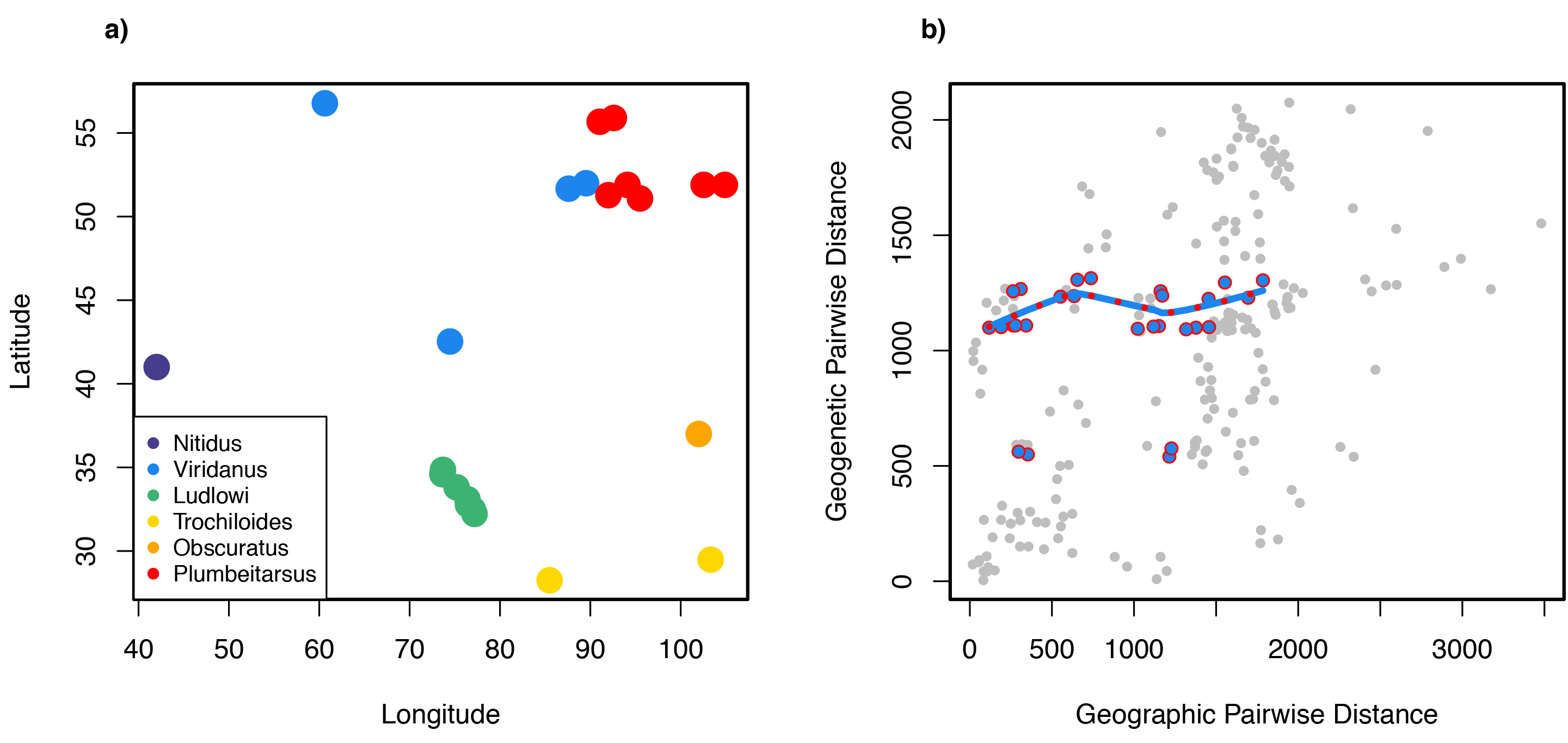

Supplement: S12 Fig — a) observed population coordinates; b) pairwise geographic (great-circle) distance between populations compared to that between their geogenetic locations. The highlighted points show distances between populations from the plumbeitarsus and viridanus subspecies. Notice that, regardless of their observed distance, their geogenetic separations are roughly constant, and much larger than the geographic distance between them. (TIF) [file pgen.1005703.s012.tif]

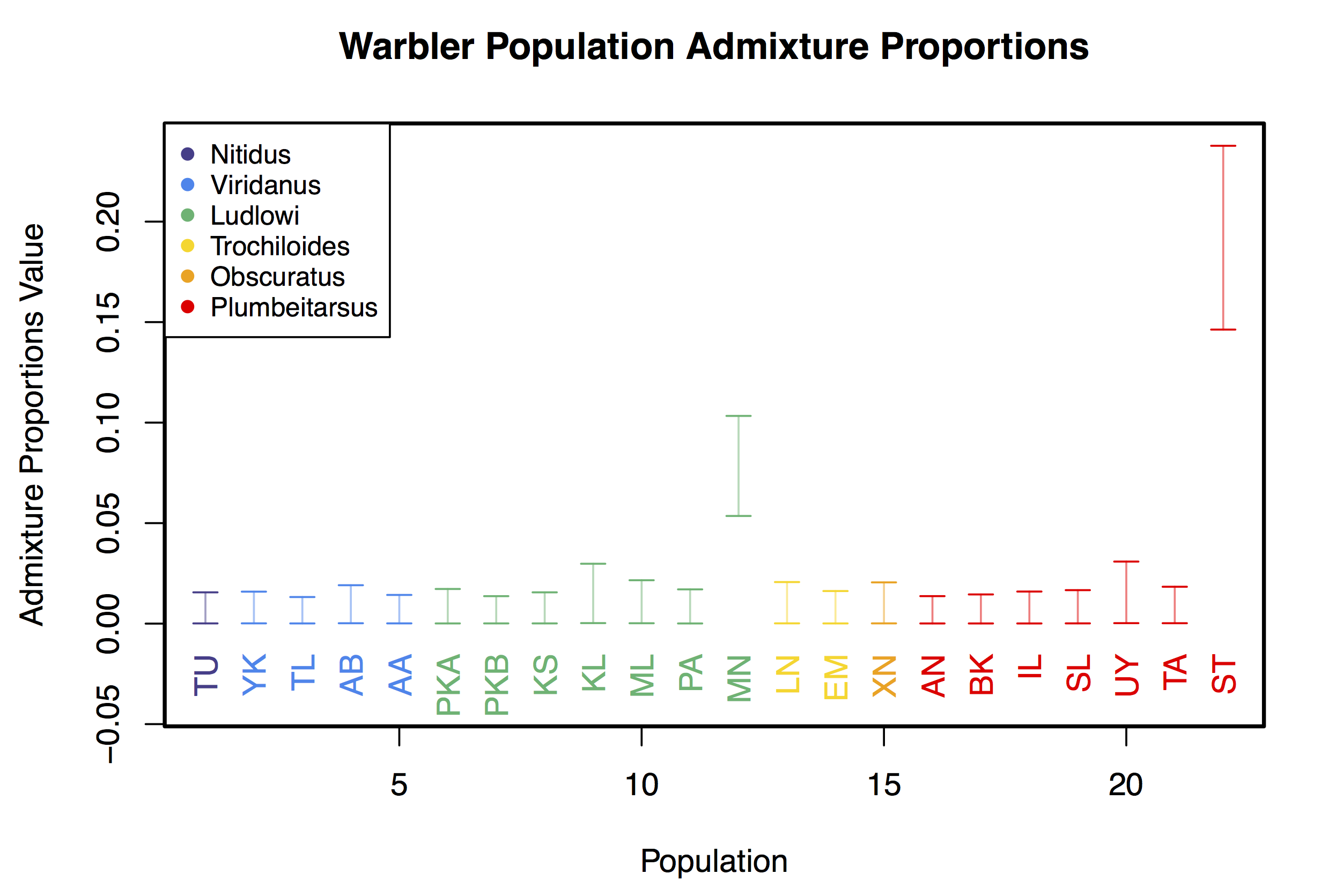

Supplement: S13 Fig — Credible intervals on estimated warbler population admixture proportion parameters. (TIF) [file pgen.1005703.s013.tif]

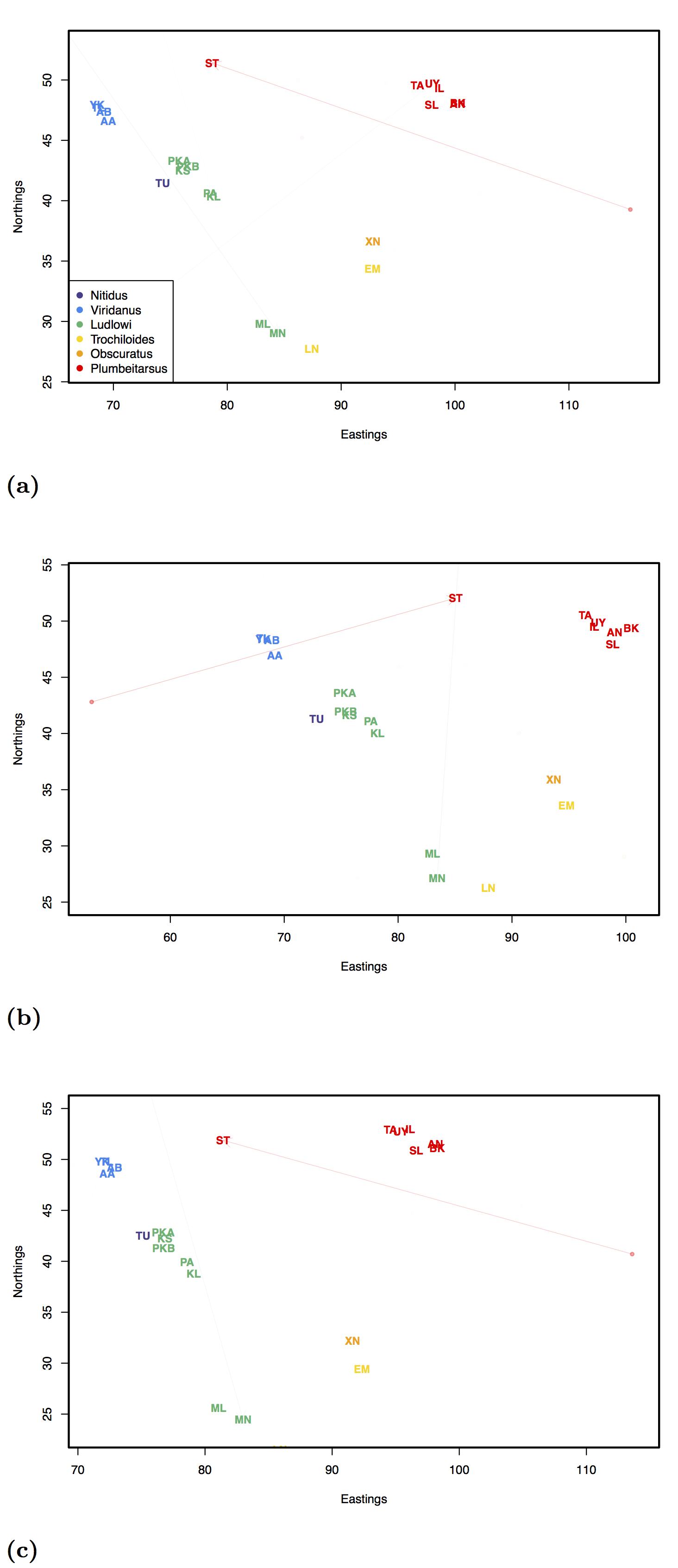

Supplement: S14 Fig — (a,b) Results from analysis using observed locations as priors on population locations. c) Results from analysis using random, uniformly distributed locations within the observed range of latitude and longitude as priors on population locations. (TIF) [file pgen.1005703.s014.tif]

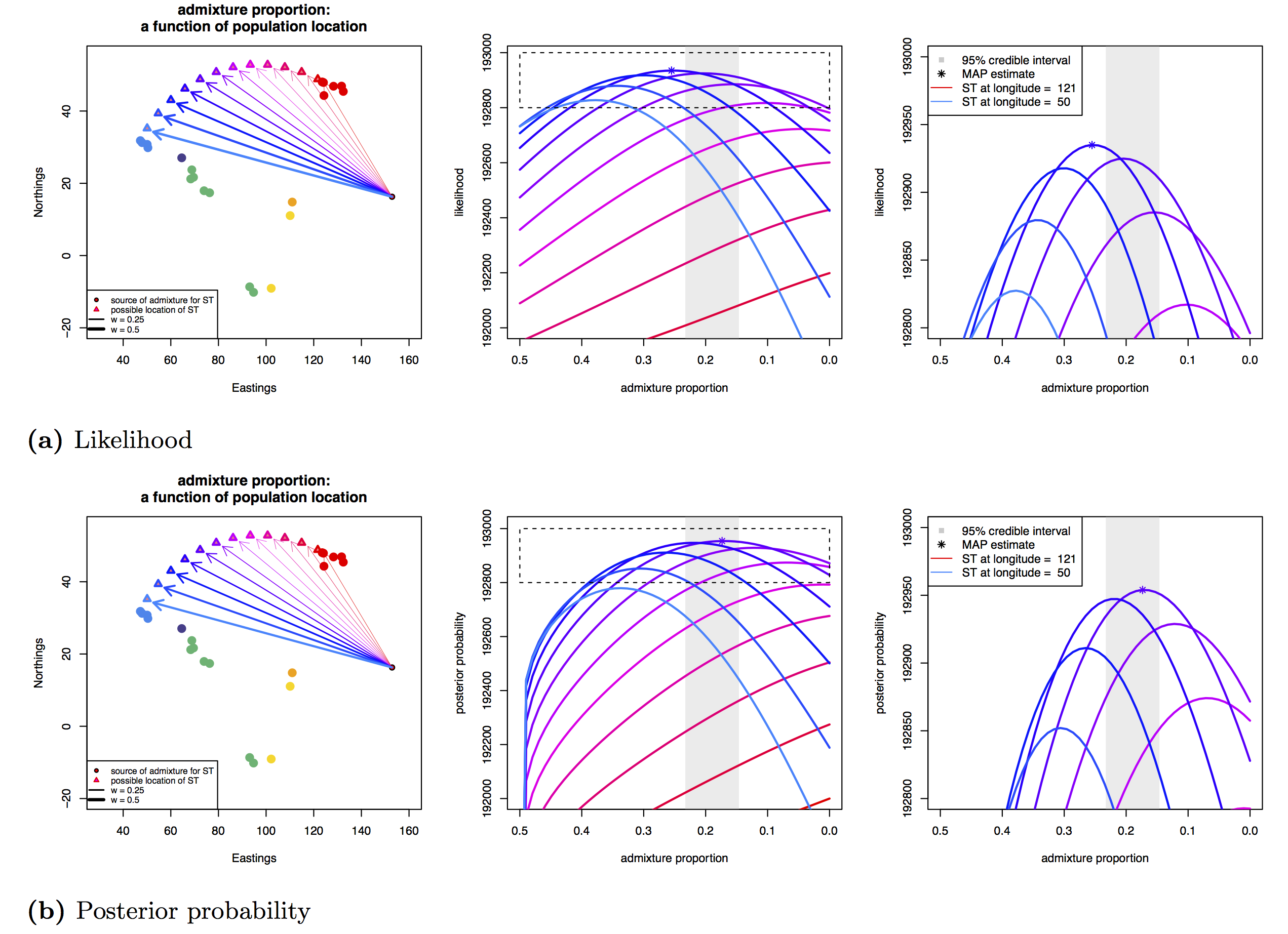

Supplement: S15 Fig — a) log likelihood surface; b) posterior probability surface, incorporating the priors. The maximum a posteriori estimate (MAP) is shown as a star. (TIF) [file pgen.1005703.s015.tif]

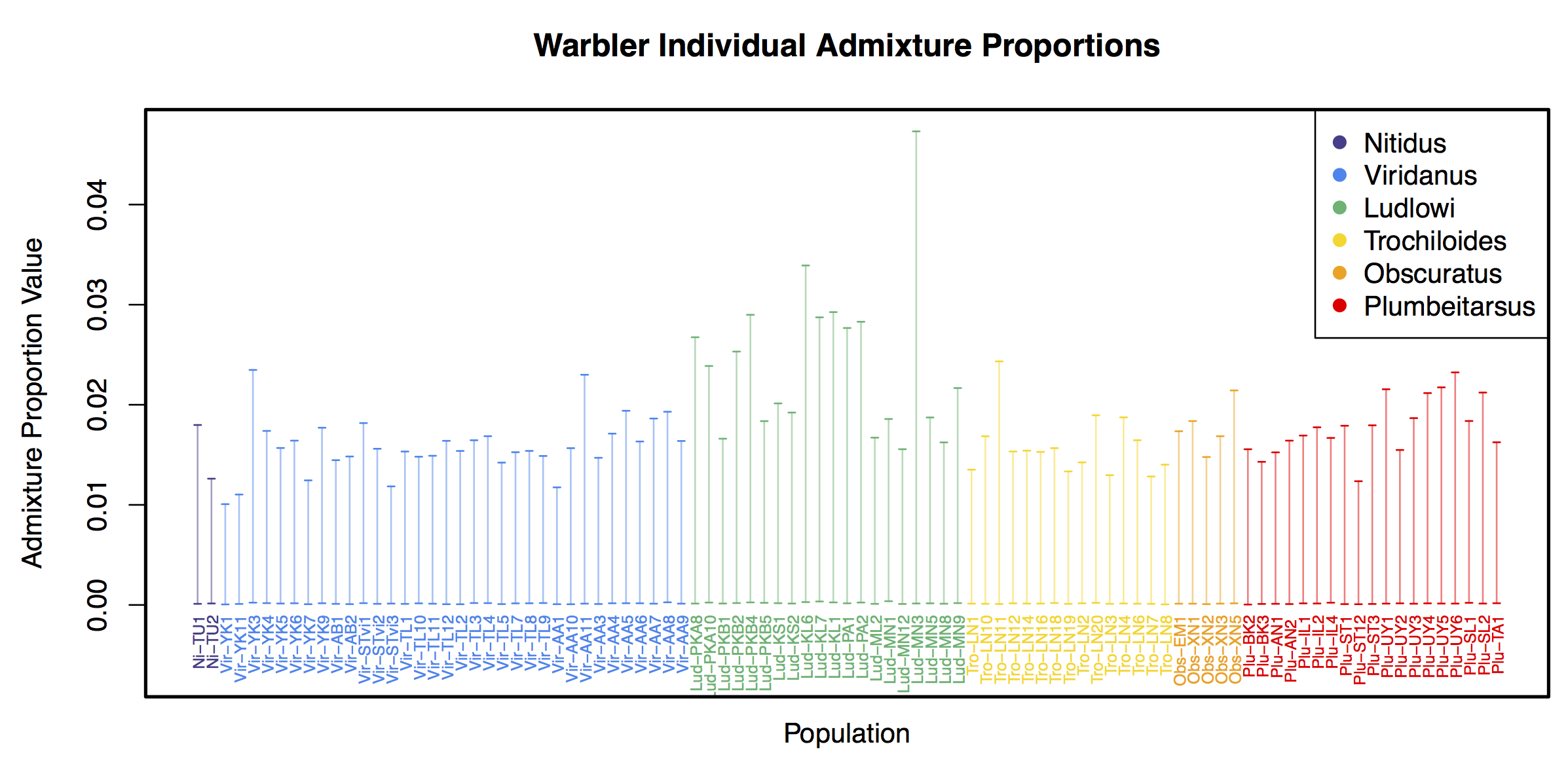

Supplement: S16 Fig — Credible intervals on estimated warbler individual admixture proportion parameters. (TIF) [file pgen.1005703.s016.tif]

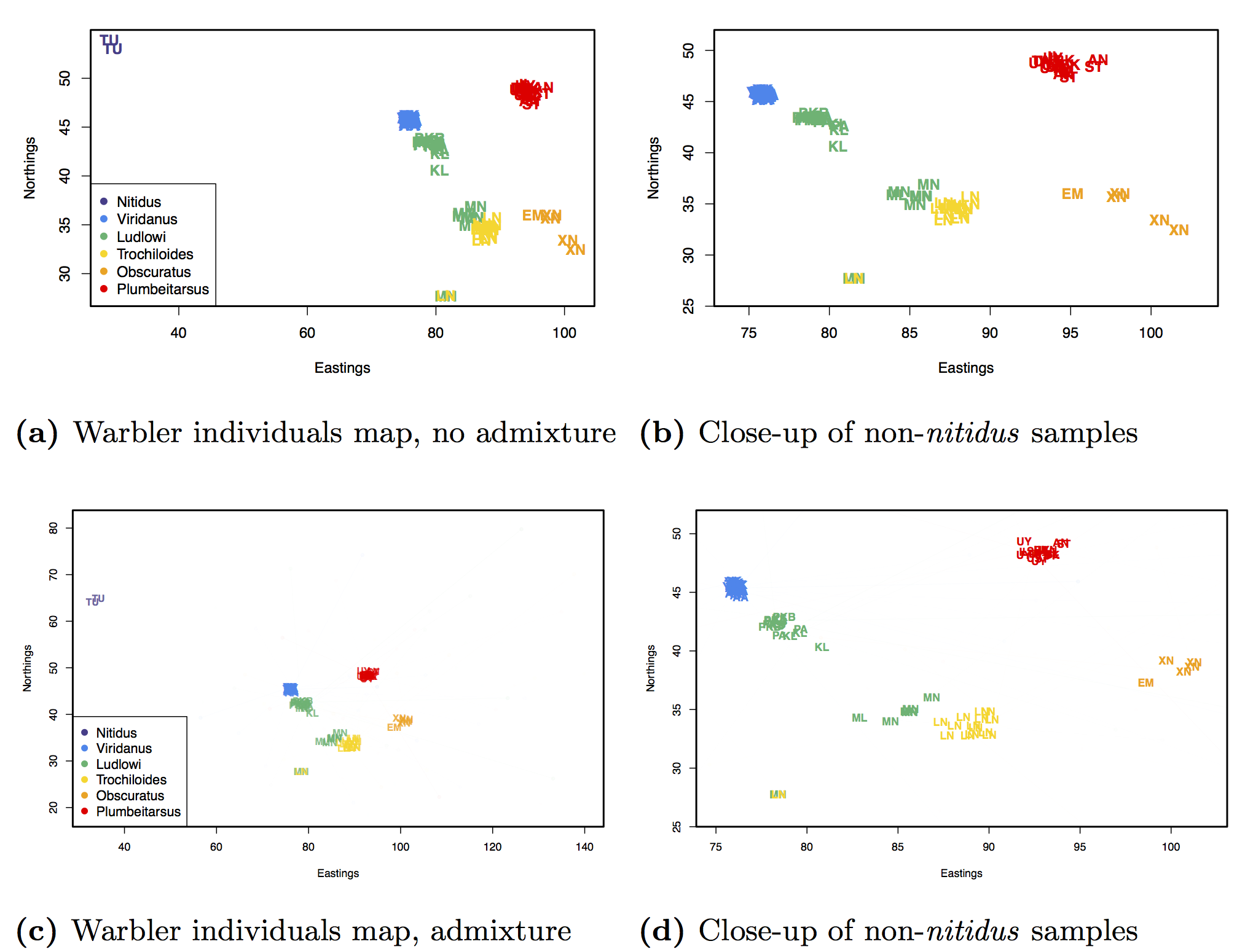

Supplement: S17 Fig — Inferred maps for warbler individuals, colored by subspecies under analyses with and without admixture inference. a) map inferred without admixture; b) close-up of all non-nitidus samples in non-admixture map; c) map inferred with admixture; d) close-up of all non-nitidus samples in the admixture map. (TIF) [file pgen.1005703.s017.tif]

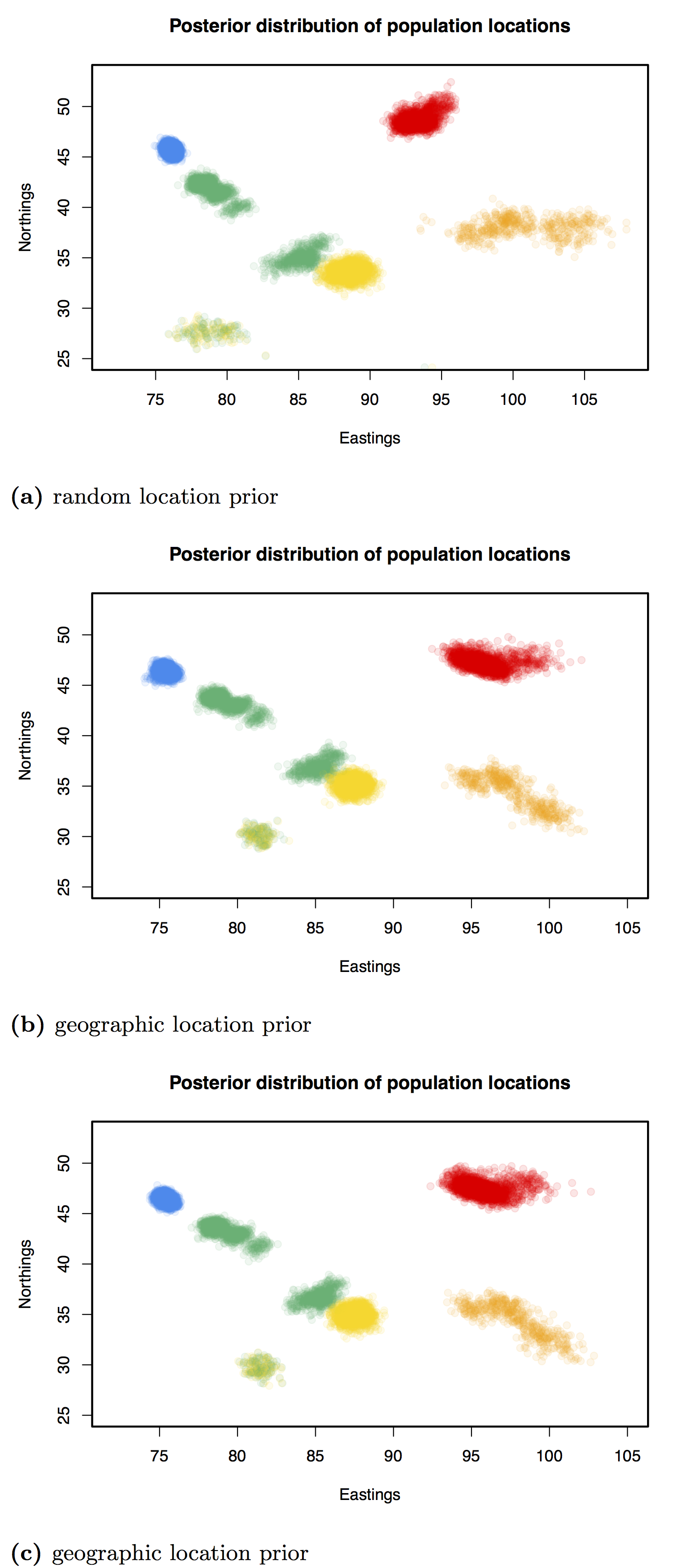

Supplement: S18 Fig — a) analysis with randomly generated priors on geogenetic location parameters; b) one analysis with true geographic locations as priors on geogenetic location parameters; c) a second analysis with true geographic locations as priors on geogenetic location parameters. (TIF) [file pgen.1005703.s018.tif]

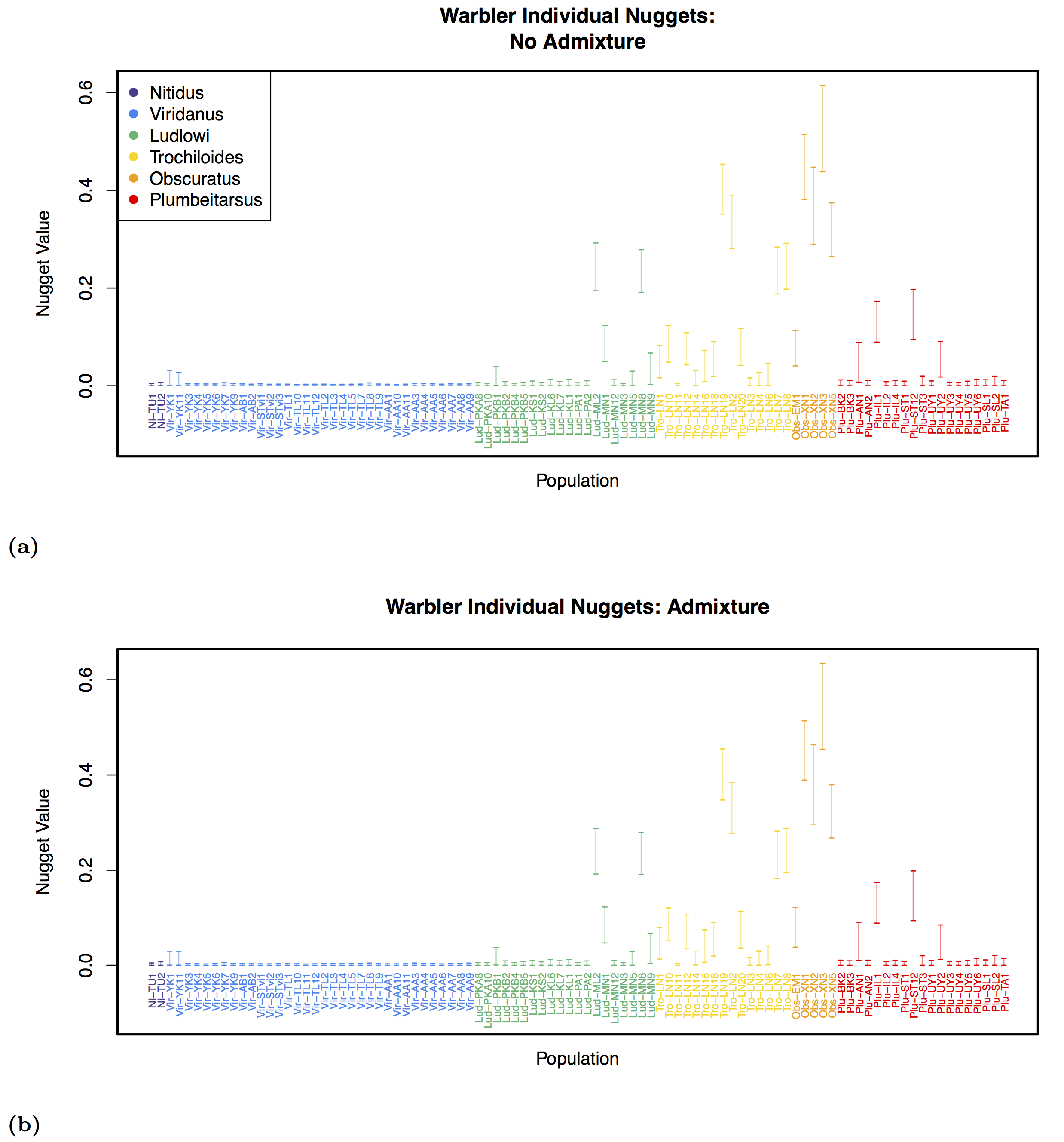

Supplement: S19 Fig — Credible intervals on estimated warbler individual nugget parameters. a) analysis without admixture; b) analysis with admixture. (TIF) [file pgen.1005703.s019.tif]

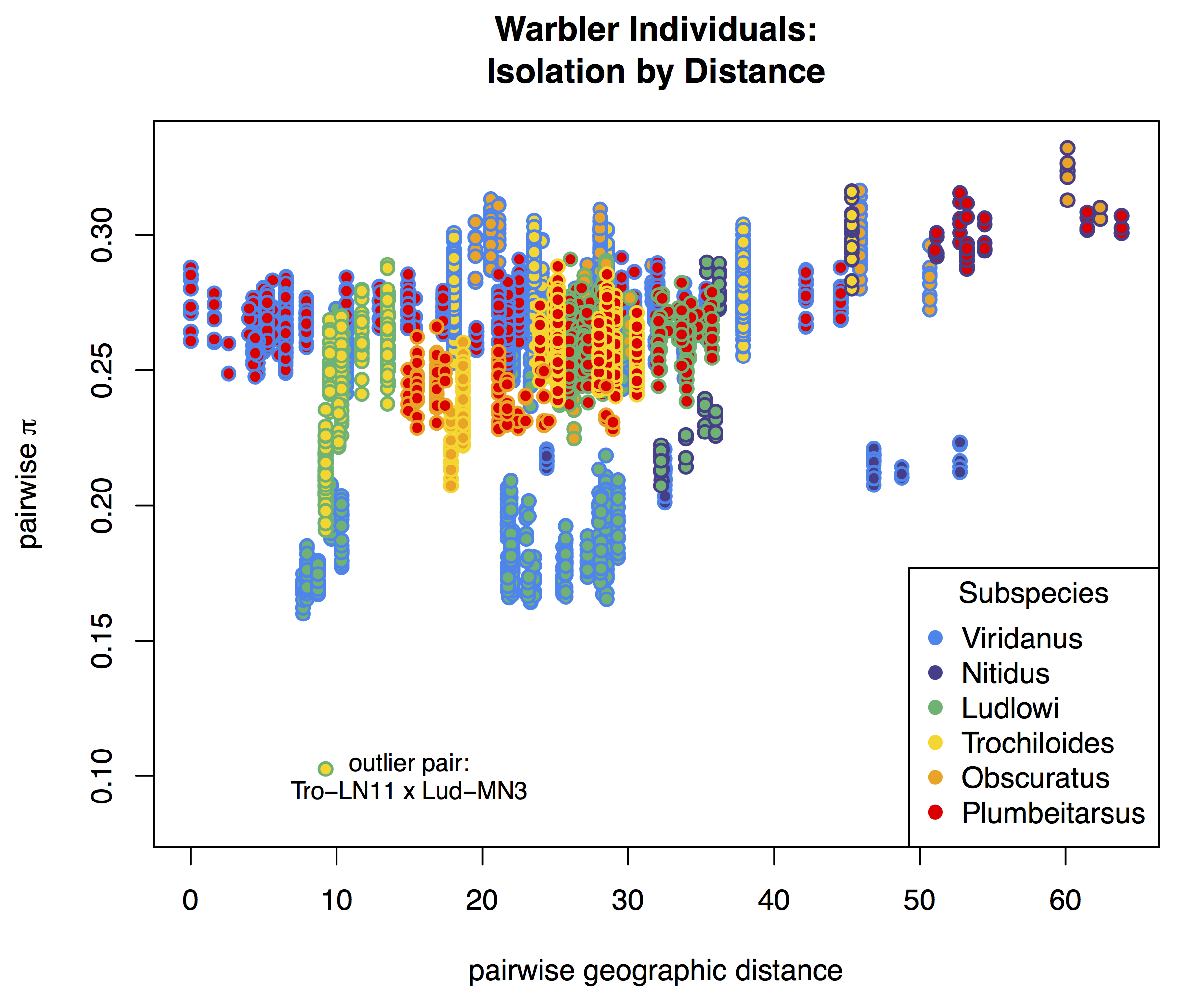

Supplement: S20 Fig — Mean pairwise sequence divergence at polymorphic sites calculated between all pairs of individuals from different subspecies, and colored by the subspecies to which each individual in the comparison is drawn. Note that individuals Tro-LN11 and Lud-MN3 have sequence divergence that is unusually low relative to that of other comparisons between individuals f rom the same two subspecies. (TIF) [file pgen.1005703.s020.tif]

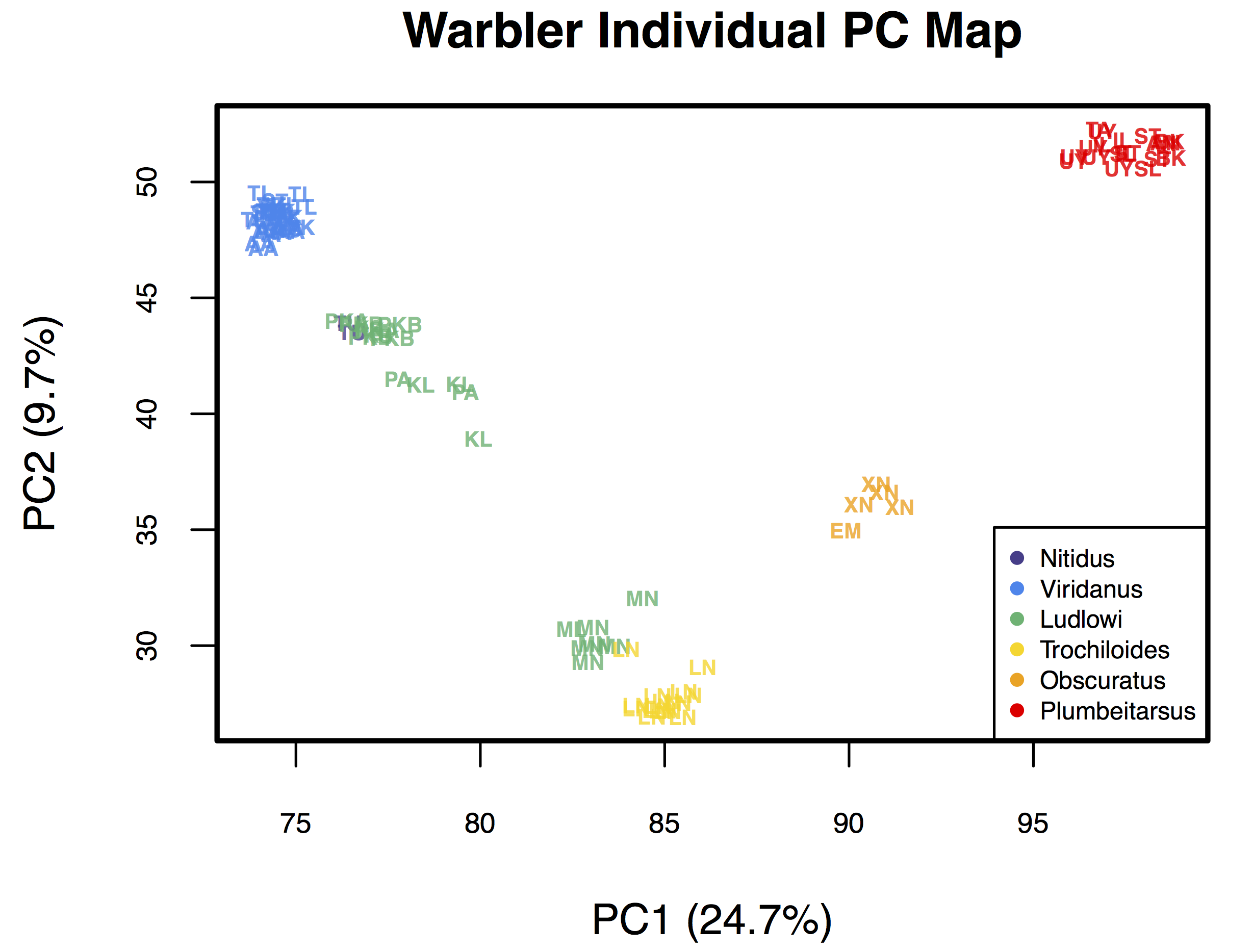

Supplement: S21 Fig — The map of warbler individuals derived from a Principal Components analysis, plotting PC1 against PC2. The PC coordinates have undergone a full Procrustes transformation around the actual sampling coordinates. (TIF) [file pgen.1005703.s021.tif]

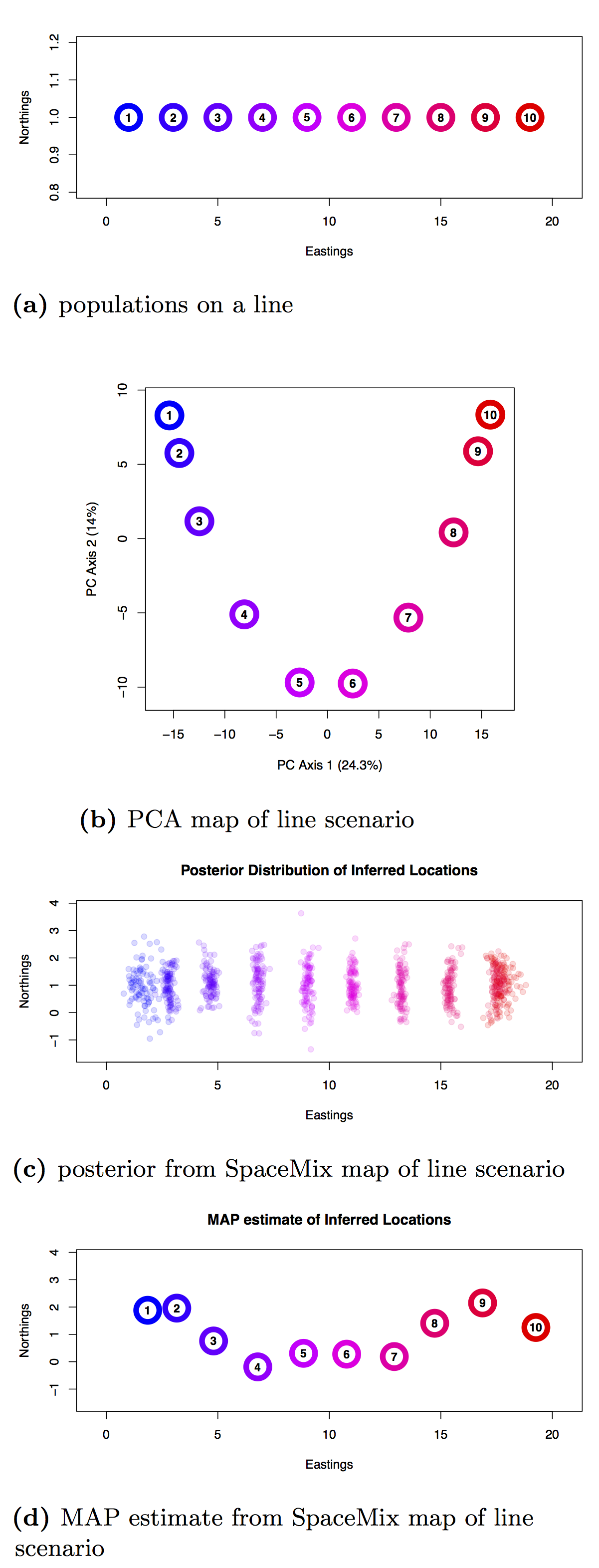

Supplement: S22 Fig — Simulation scenario of populations on a line, contrasting PCA-based inference and SpaceMix inference. a) Scenario used to simulate data in a spatial coalescent framework with nearest-neighbor migration; b) PCA map of allele frequencies, plotting PC axis 1 against PC axis 2, forming a ‘U’ shape; c) Posterior distribution of SpaceMix location inference, forming a rough line; d) Snapshot of the MAP draw from the posterior, again showing a rough line. (TIF) [file pgen.1005703.s022.tif]

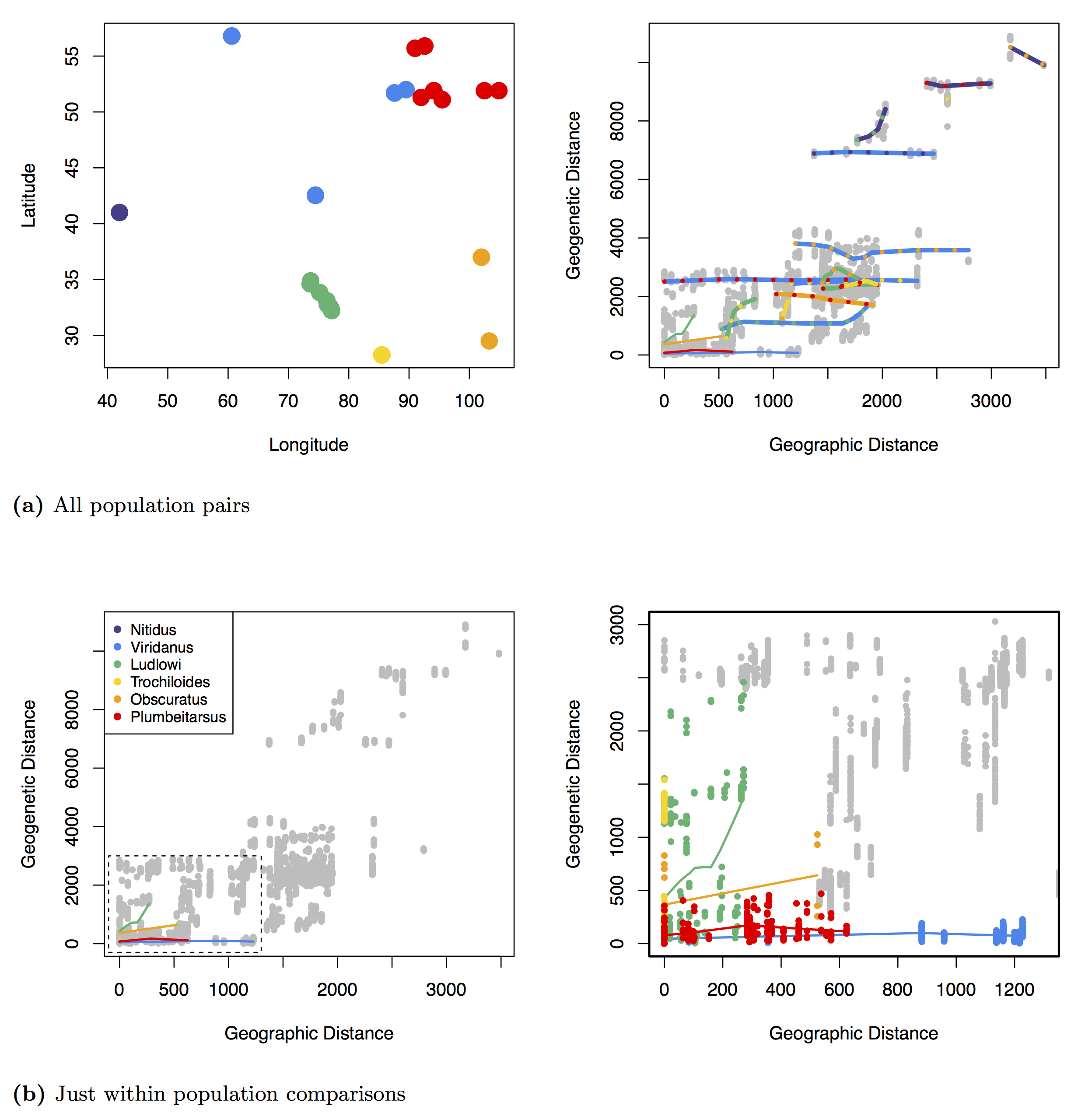

Supplement: S23 Fig — a) comparisons between populations in different subspecies. b) comparisons between populations in the same subspecies. (TIF) [file pgen.1005703.s023.tif]

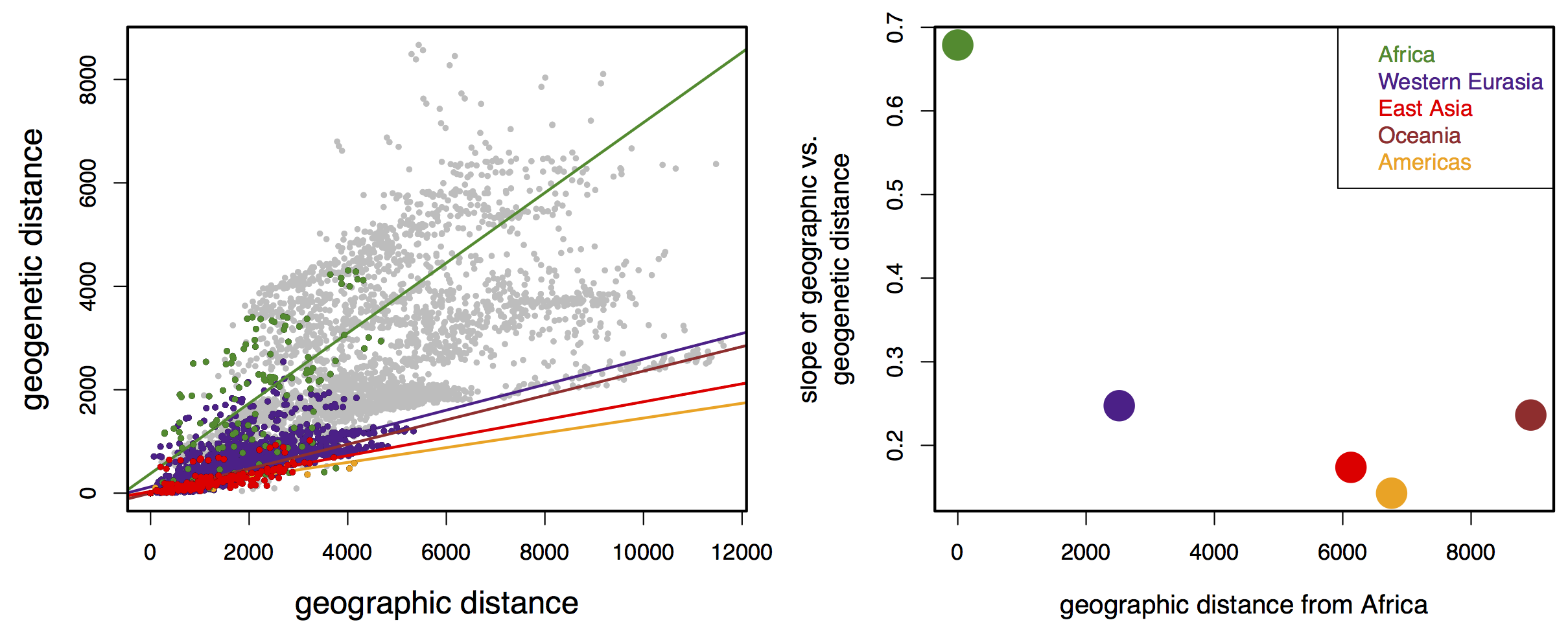

Supplement: S24 Fig — Comparisons are colored by continent from which populations were sampled (i.e., two populations sampled from Africa are green). Eurasia is divided into Western Eurasia and East Asia. (TIF) [file pgen.1005703.s024.tif]

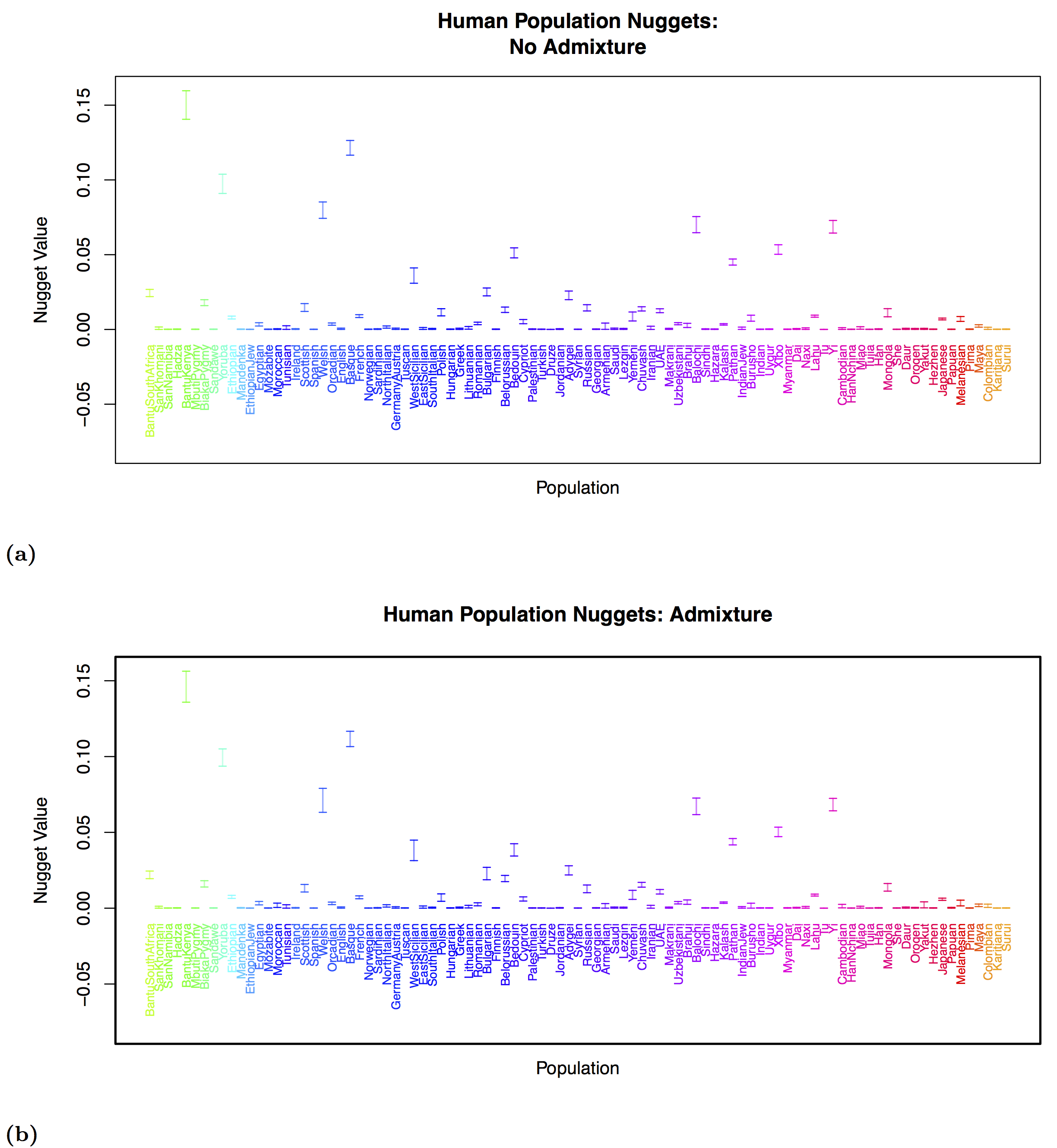

Supplement: S25 Fig — a) analysis without admixture; a) analysis with admixture. (TIF) [file pgen.1005703.s025.tif]

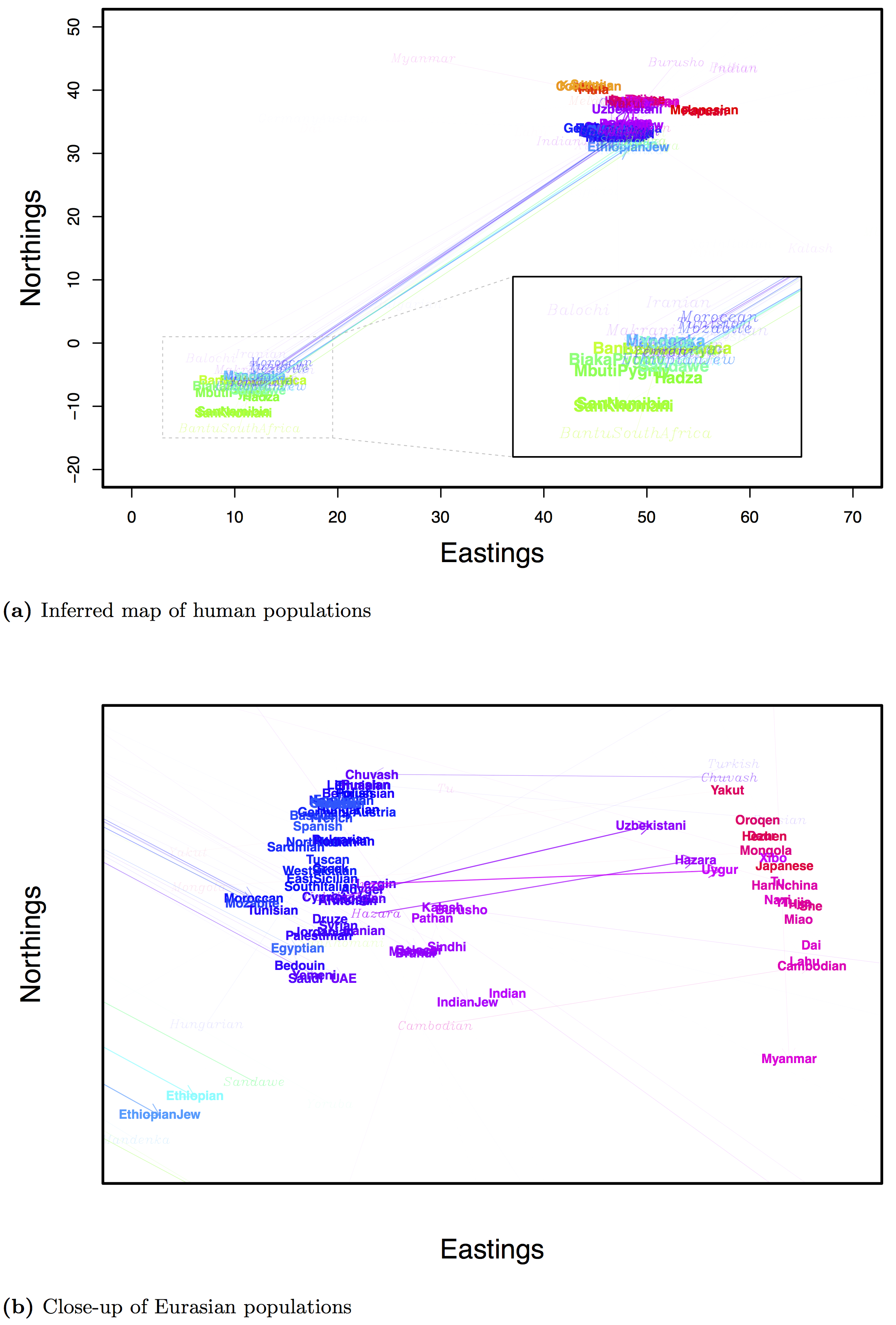

Supplement: S26 Fig — Map of human populations from a different SpaceMix analysis than that reported in the main text (“Real_Prior1”—inferred with admixture), using real geographic coordinates as population location priors. a) complete map; b) close-up of Eurasian populations. (TIF) [file pgen.1005703.s026.tif]

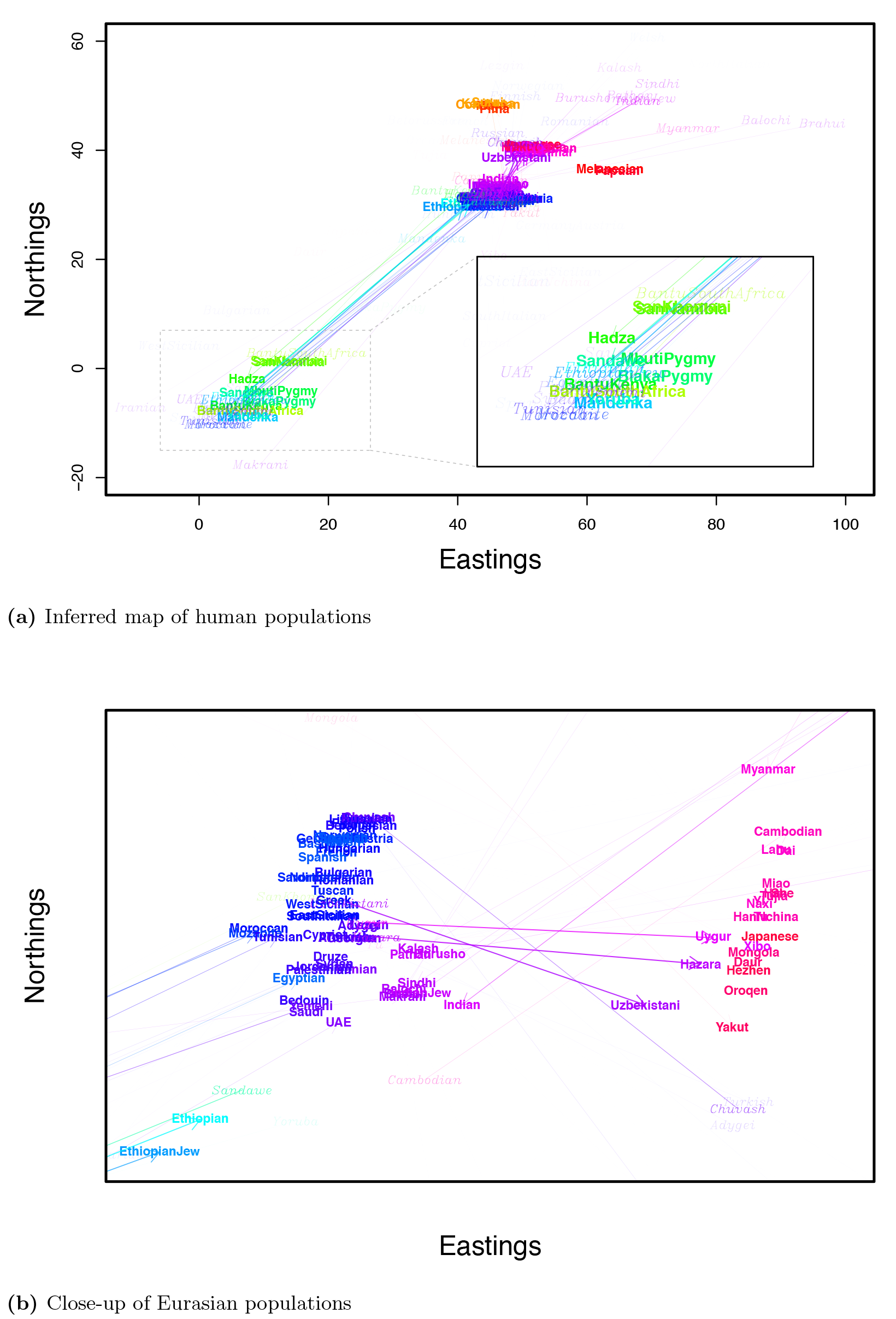

Supplement: S27 Fig — Map of human populations from another SpaceMix analysis (“Real_Prior2”, inferred with admixture), using real geographic coordinates as population location priors. a) complete map; b) close-up of Eurasian populations. (TIF) [file pgen.1005703.s027.tif]

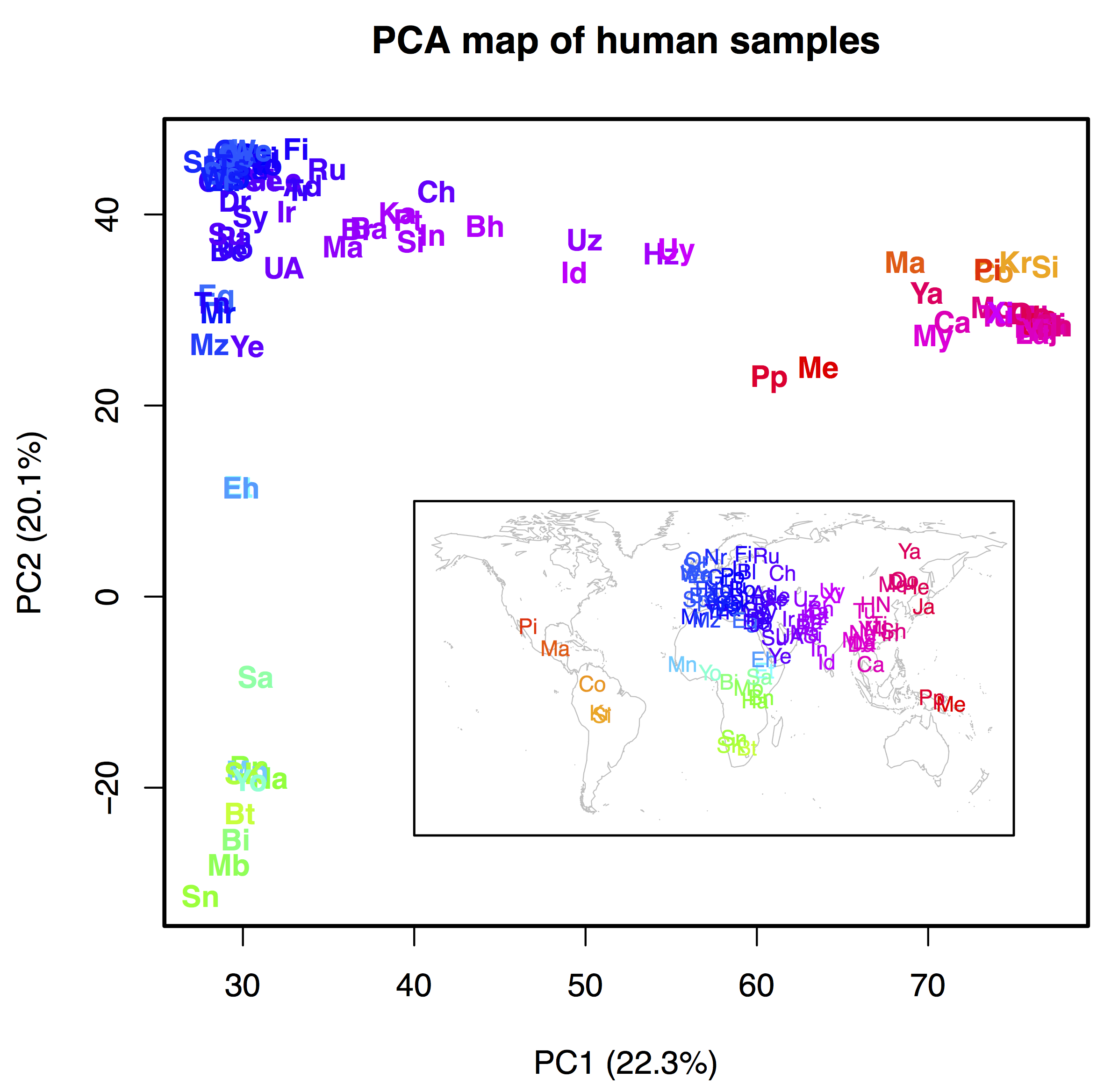

Supplement: S28 Fig — The PC coordinates have undergone a full Procrustes transformation around the actual sampling coordinates (shown in the inset map). (TIF) [file pgen.1005703.s028.tif]

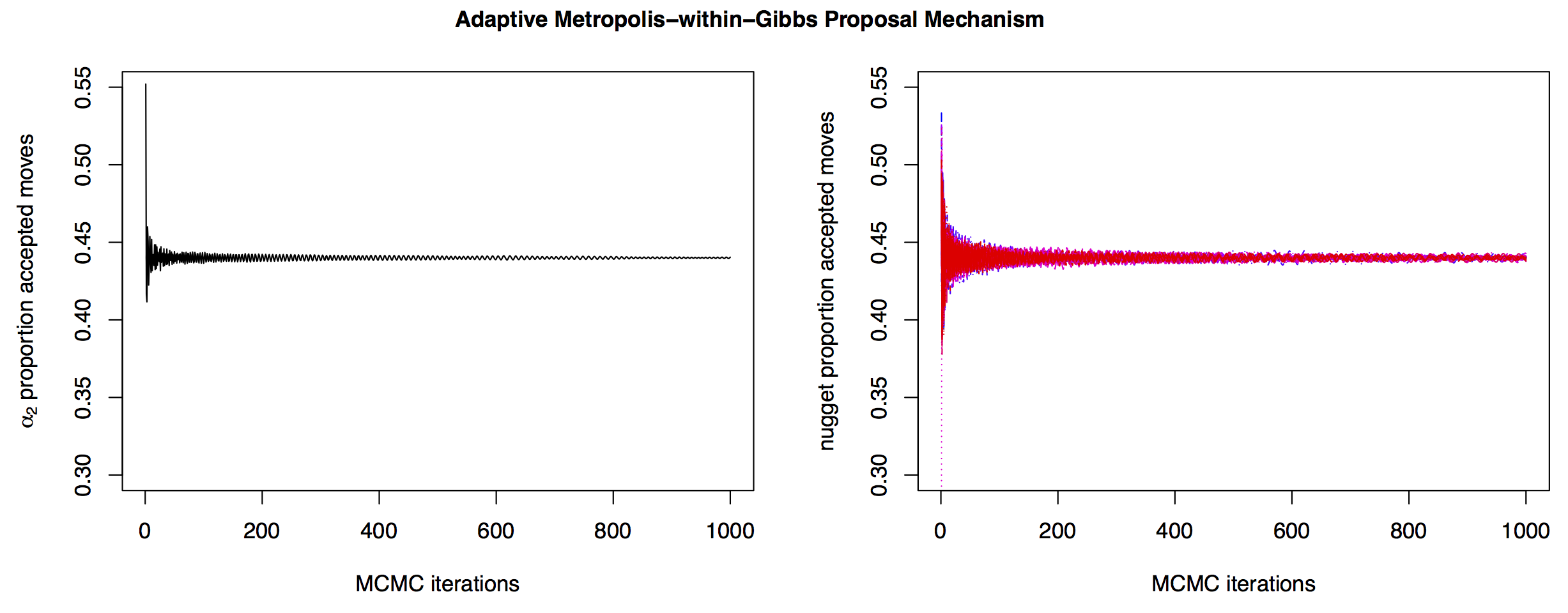

Supplement: S29 Fig — Example parameter acceptance proportions for the α2 parameter and the nugget parameter, η, using the adaptive Metropolis-within-Gibbs proposal mechanism. (TIF) [file pgen.1005703.s029.tif]

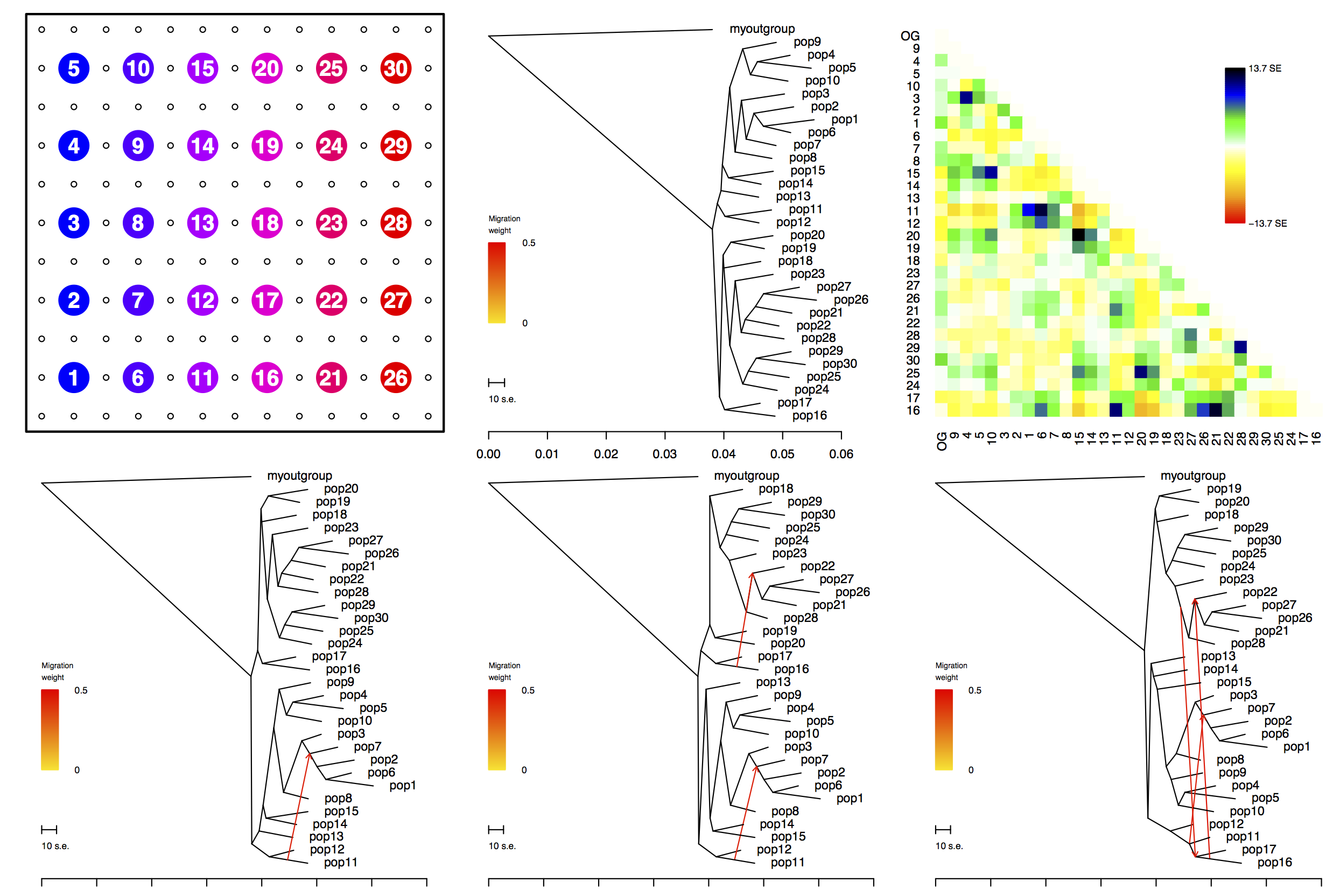

Supplement: S30 Fig — The tree, residual covariance matrix, and first three migration admixture arrows are shown. (TIF) [file pgen.1005703.s030.tif]

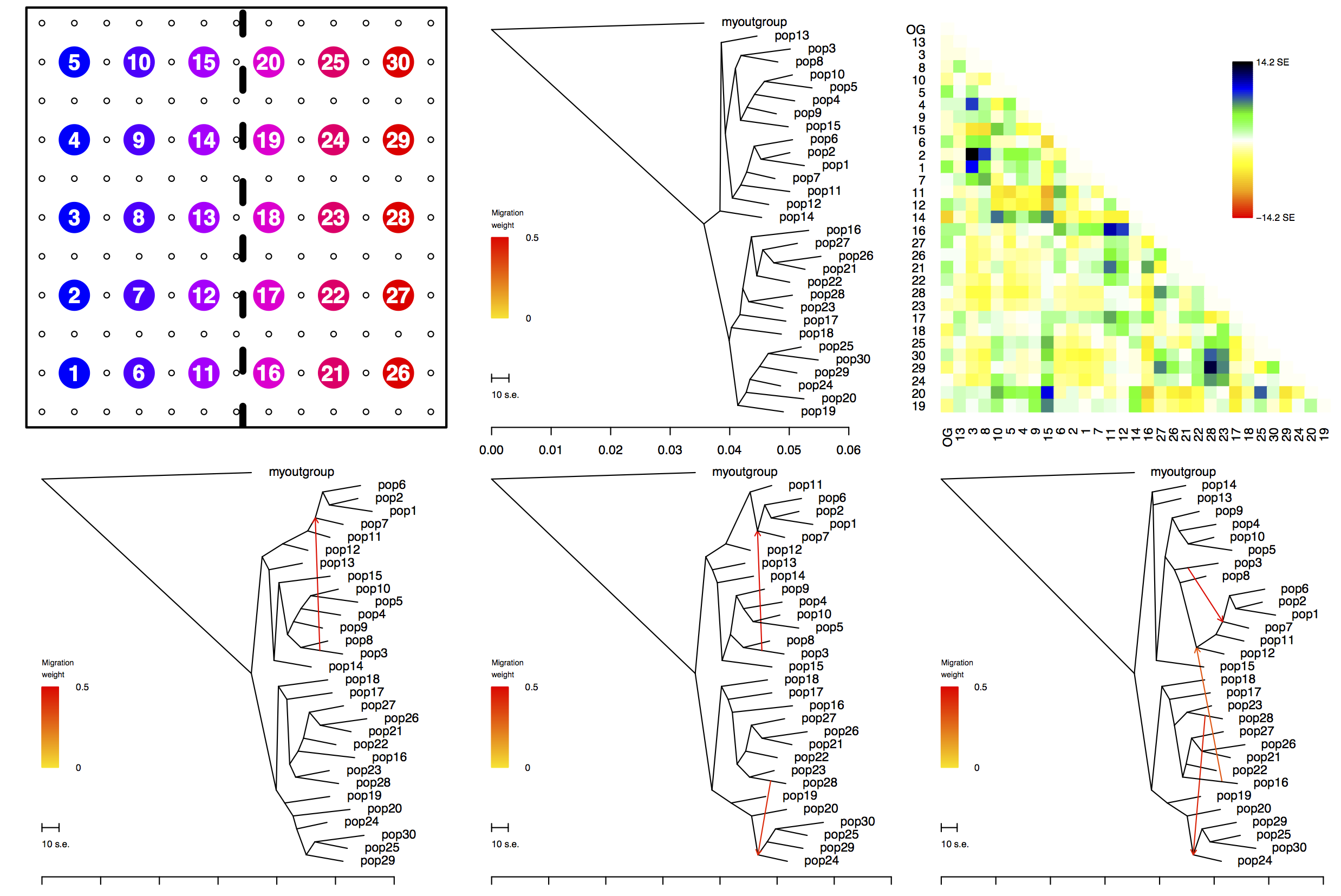

Supplement: S31 Fig — The tree, residual covariance matrix, and first three migration admixture arrows are shown. (TIF) [file pgen.1005703.s031.tif]

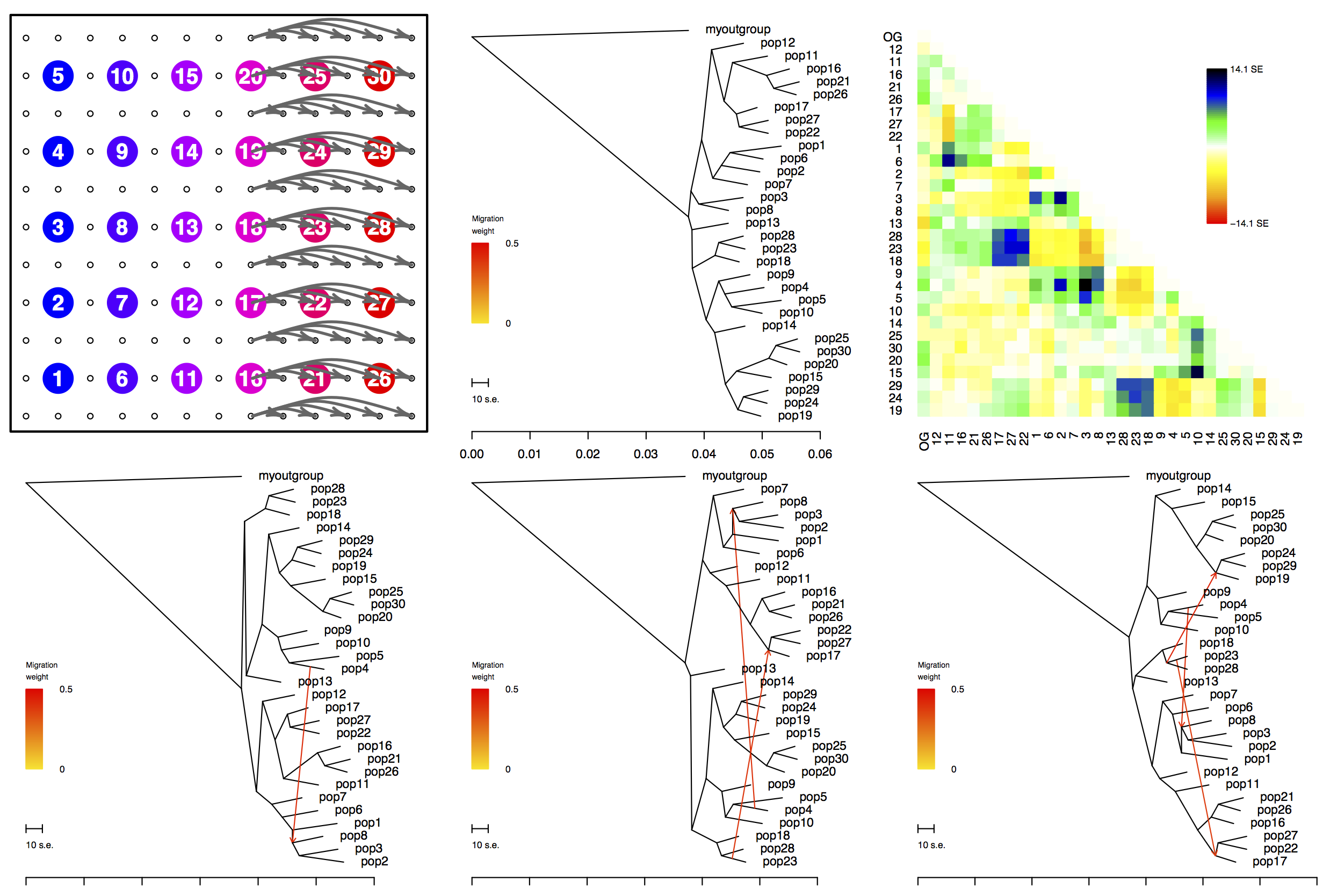

Supplement: S32 Fig — The tree, residual covariance matrix, and first three migration admixture arrows are shown. (TIF) [file pgen.1005703.s032.tif]

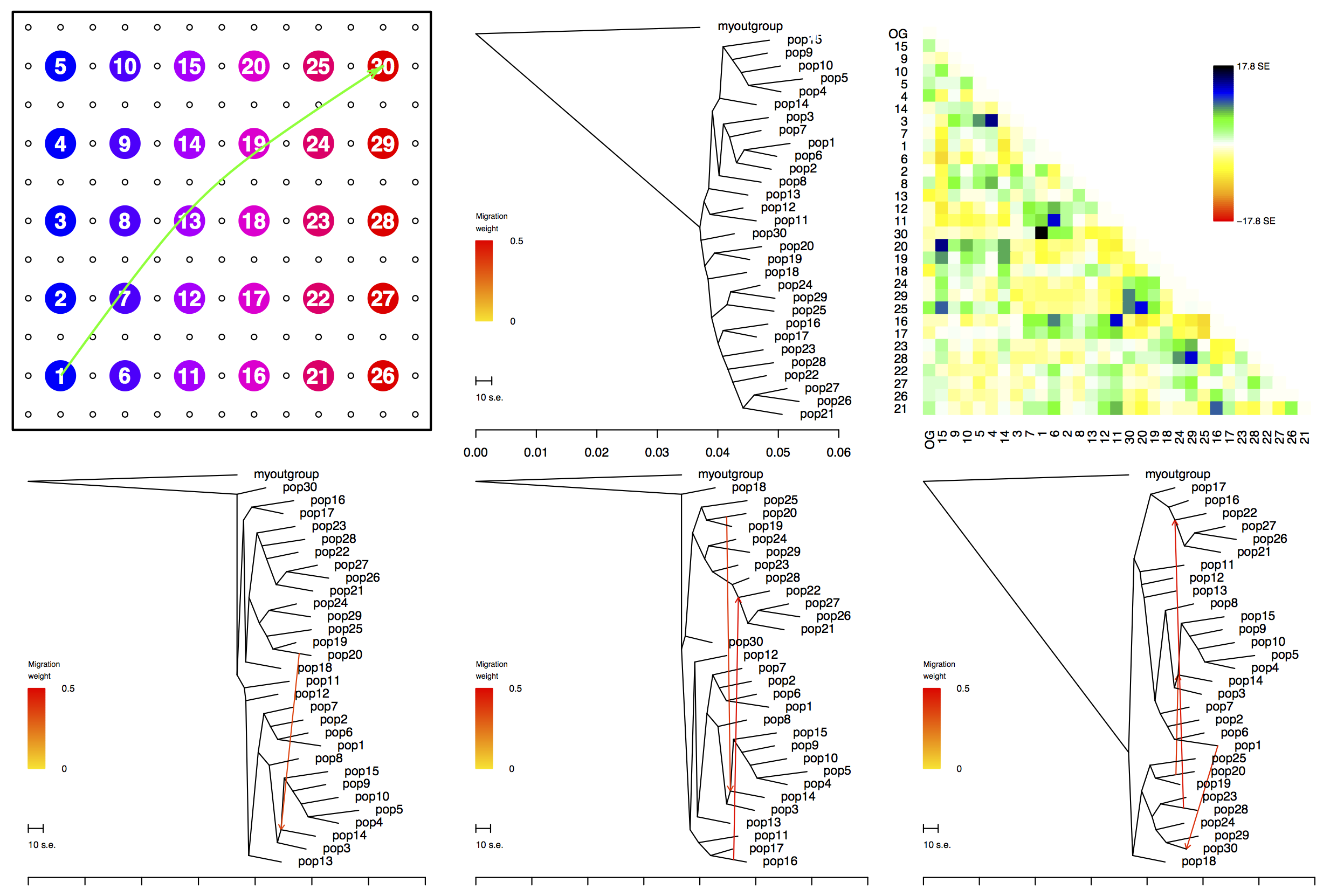

Supplement: S33 Fig — The tree, residual covariance matrix, and first three migration admixture arrows are shown. (TIF) [file pgen.1005703.s033.tif]

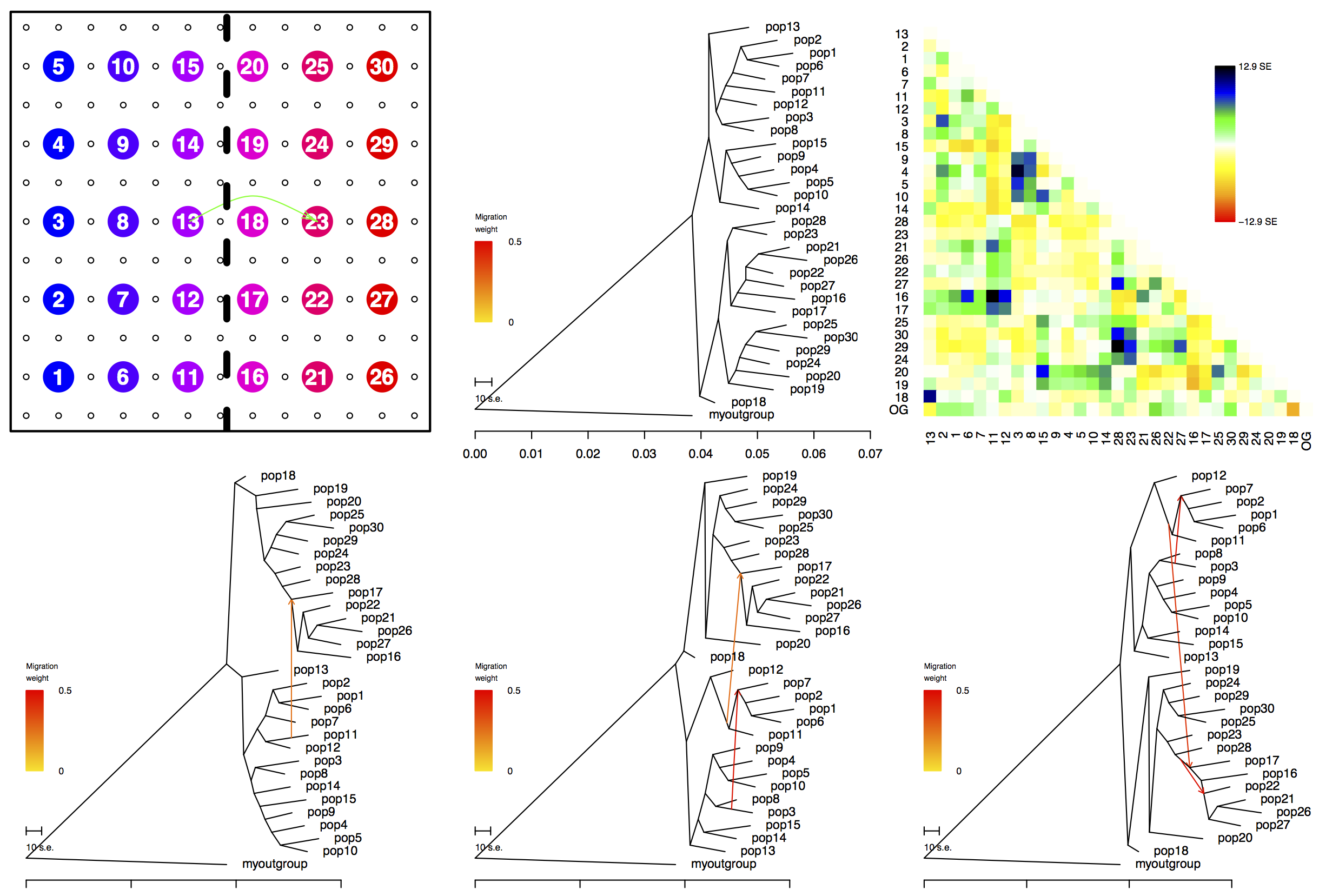

Supplement: S34 Fig — The tree, residual covariance matrix, and first three migration admixture arrows are shown. (TIF) [file pgen.1005703.s034.tif]

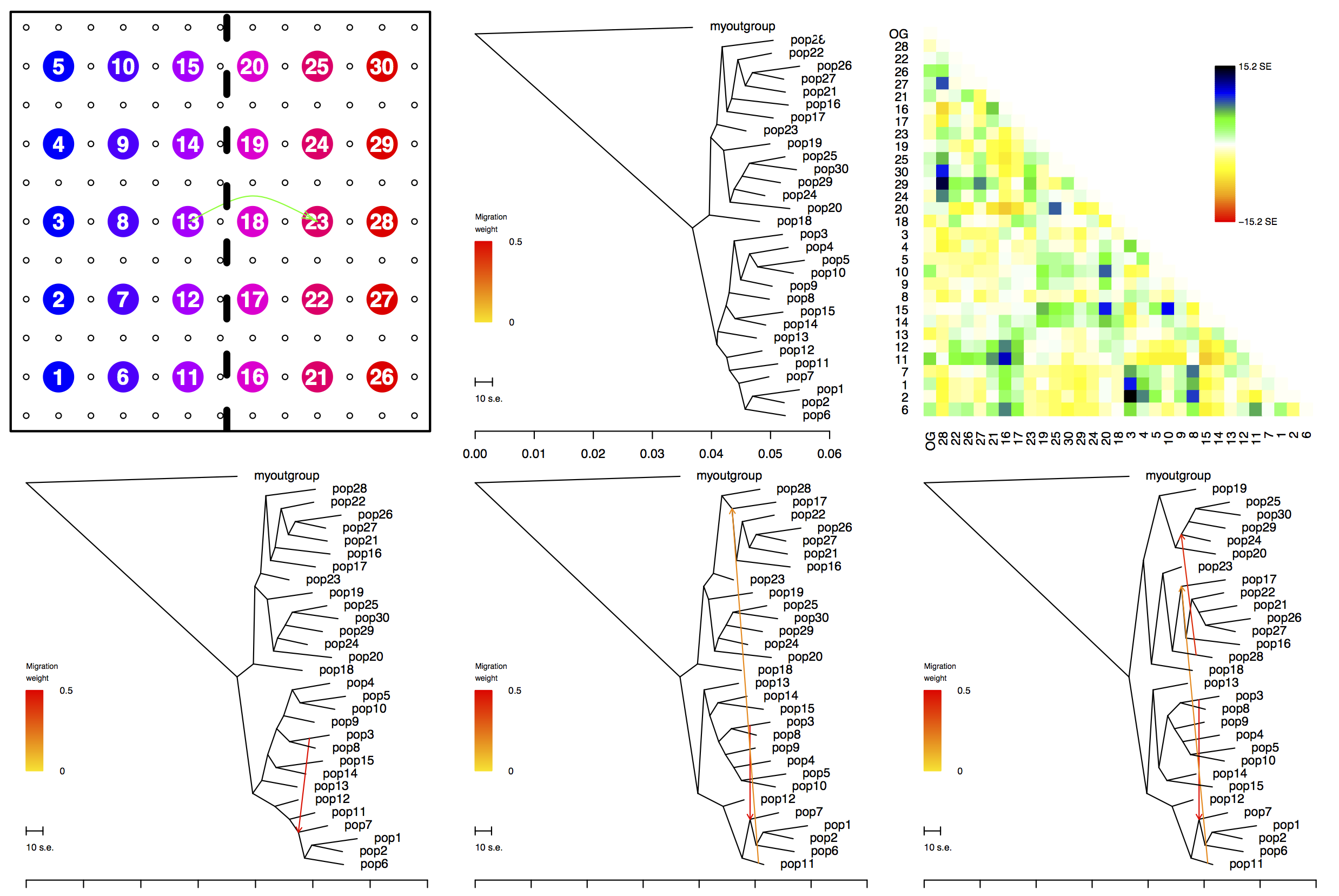

Supplement: S35 Fig — The tree, residual covariance matrix, and first three migration admixture arrows are shown. (TIF) [file pgen.1005703.s035.tif]

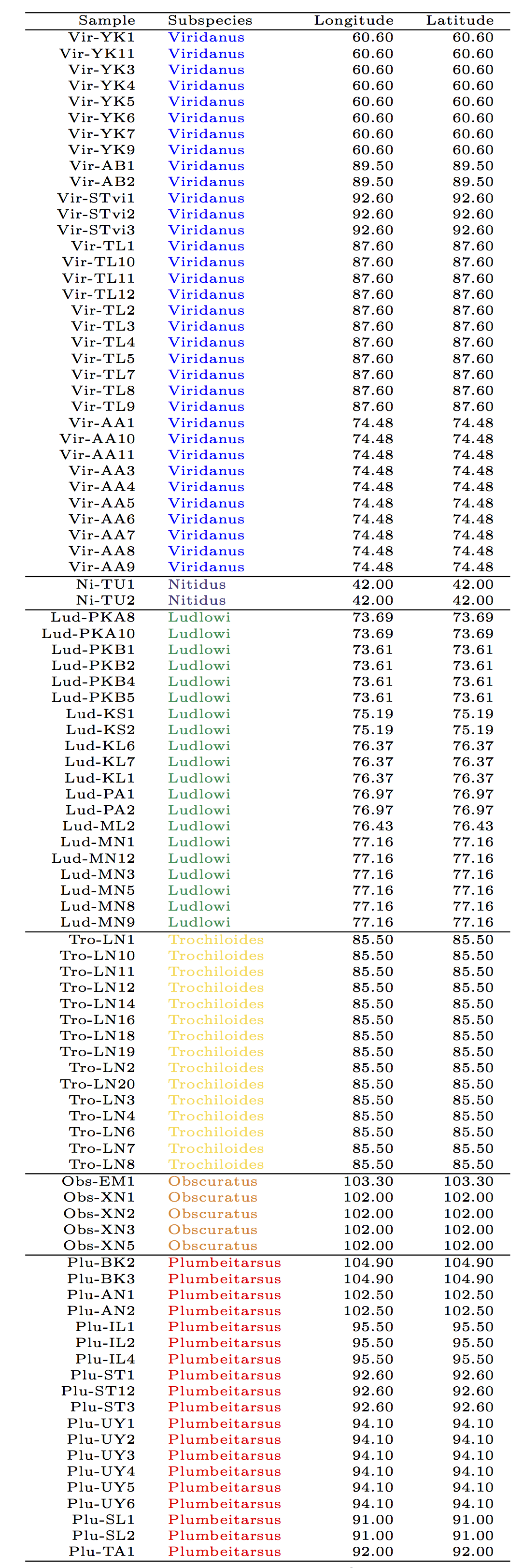

Supplement: S1 Table — Subspecies and geographic meta-data for greenish warbler individuals included in analysis. (TIF) [file pgen.1005703.s036.tif]

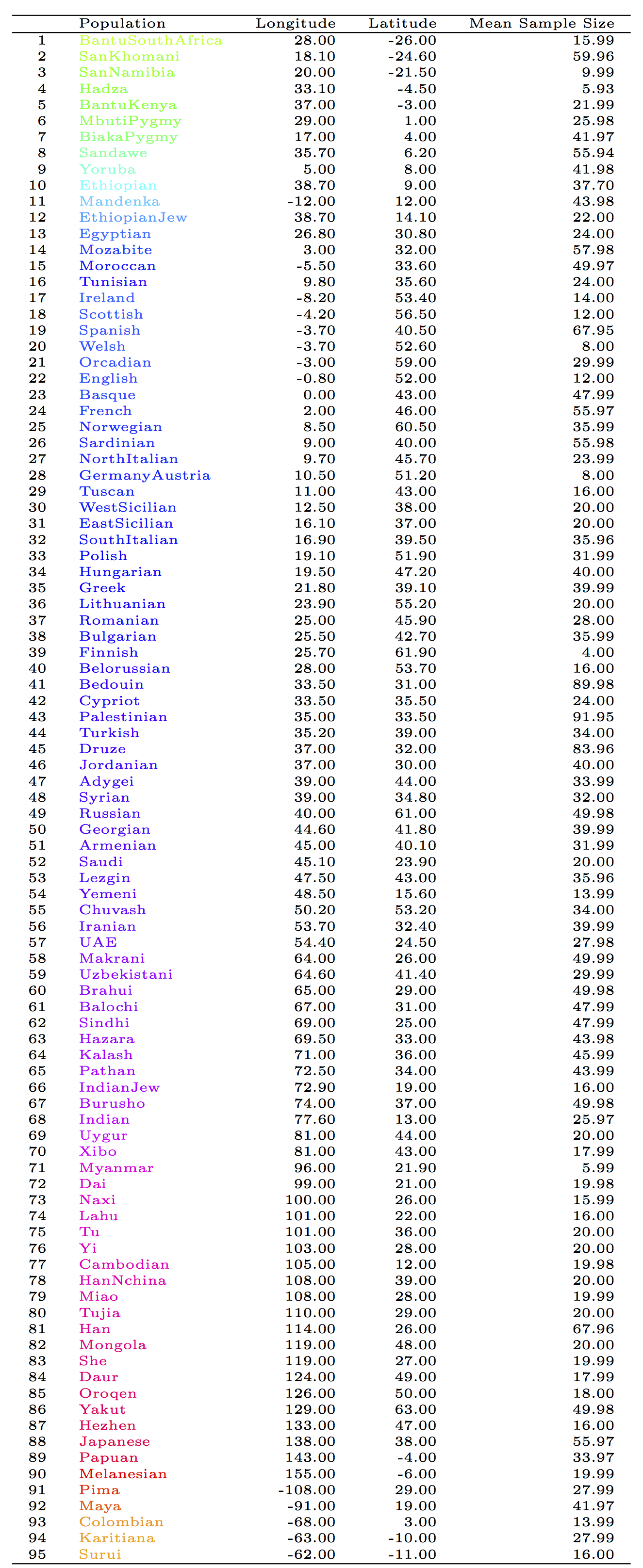

Supplement: S2 Table — Sample size and geographic meta-data for human samples included in analysis. (TIF) [file pgen.1005703.s037.tif]
